# Study on micro crack propagation mechanism of ferrite–pearlite gas transmission pipeline steel with lamellar structure

**DOI:** 10.1038/s41598-022-23405-4

**Published:** 2022-11-04

**Authors:** Taolong Xu, Wei Wang, Hongye Jiang, Gongzhen He

**Affiliations:** 1 Petroleum Engineering School, Southwest Petroleum University, Chengdu, 610500 Sichuan China; 2 Guangzhou Urban Management Quality Monitoring and Emergency Support Center (Urban Gas Affairs Center of Guangzhou City), Guangzhou, 510623 Gangdong China; 3 Sichuan East Gas Transmission Sales Center of Sinopec Natural Gas Branch, Wuhan, 430074 Hubei China

**Keywords:** Engineering, Materials science

## Abstract

The deformation and failure characteristics of pipeline steel depend on its atomic structure and microstructure. Based on the serial multi-scale analysis technology, the ferrite/cementite (α-Fe/Fe_3_C) lamellar atomic structure with Bagaryatskii orientation relationship is established. In order to obtain the experimental sample of the lowest energy state, The step-by-step relaxation method of conjugate gradient energy minimization and constant temperature and constant pressure relaxation under NPT conditions is carried out, and the energy state and atomic structure of the relaxed samples are analyzed. For the models of different cementite terminal plane structures, the tension displacement curves on the propagation path of mode I central through crack are extracted respectively, combined with the bilinear cohesion zone model, The cohesion parameters at the atomic scale are successfully transferred from bottom to top to the macro and micro scales. By simulating the reaction force and displacement response law at the loading point, the critical fracture toughness of each terminal interface of ferrite–pearlite pipeline steel at different scales is calculated, which provides a reliable path for exploring the micro mechanism of macro cracking behavior of pipeline steel.

## Introduction

In recent years, many fracture, combustion and explosion accidents have occurred in large gas transmission pipelines all over the world. The research on the failure mechanism and ontology risk prevention and control of pipeline steel has become one of the main problems in the engineering field. The essence of everything can only be grasped by investigating its root, and finding the internal law and mechanism of microcrack propagation process and the coupling relationship between defect initiation critical load and microstructure are the only way to improve the safety of pipeline body. Pipeline steel fracture is a multi-level process combining macro and micro. Therefore, with the help of material serial multi-scale analysis technology, this study will try to build a link between micro defect initiation and macro damage of pipeline steel, so as to provide new ideas for solving the interdisciplinary scientific problems in the intrinsic safety of gas transmission pipeline, so as to avoid the heavy losses caused by pipeline fracture to the society.

**Figure 1:**
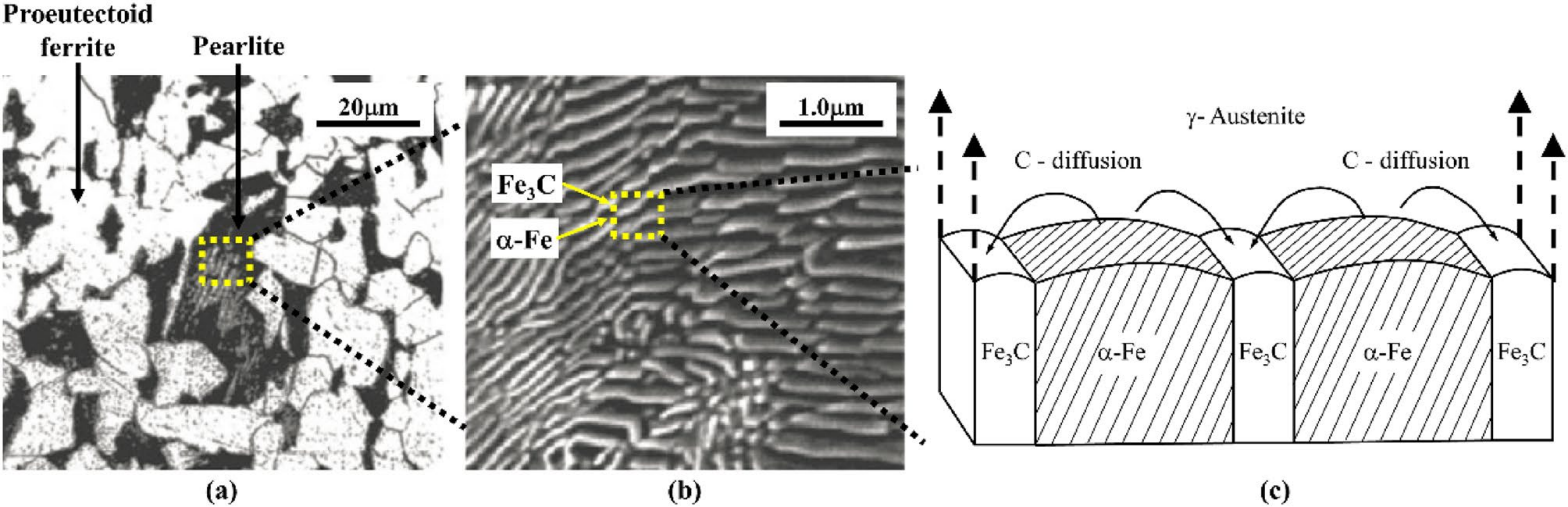
Microstructure of ferrite–pearlite steel. (**a**) Ferrite grains and pearlite aggregates. (**b**) Lamellar pearlite aggregate. (**c**) Schematic diagram of ferrite/cementite lamella structure.

Statistics show that the fracture failure of ferrite–pearlite pipeline steel mainly comes from stress corrosion cracking (SCC), corrosion fatigue (CF) and hydrogen induced cracking (HIC)^1^. When these environmentally assisted cracking (EAC) are in the uncertain load environment inside and outside the pipeline, the defects may continue to develop. In the past decades, the typical structure of high strength and toughness pipeline steel has always been ferrite (α-Fe)-pearlite (Fe_3_C)^2^ (as shown in Fig. 1a). The ferrite strength is low, but it can withstand large plastic deformation, the pearlite strength is high, and has a great impact on the plastic brittle transformation characteristics of pipeline steel, because it has ferrite/cementite lamellar structure as shown in Fig. 1b,c.

**Figure 2:**
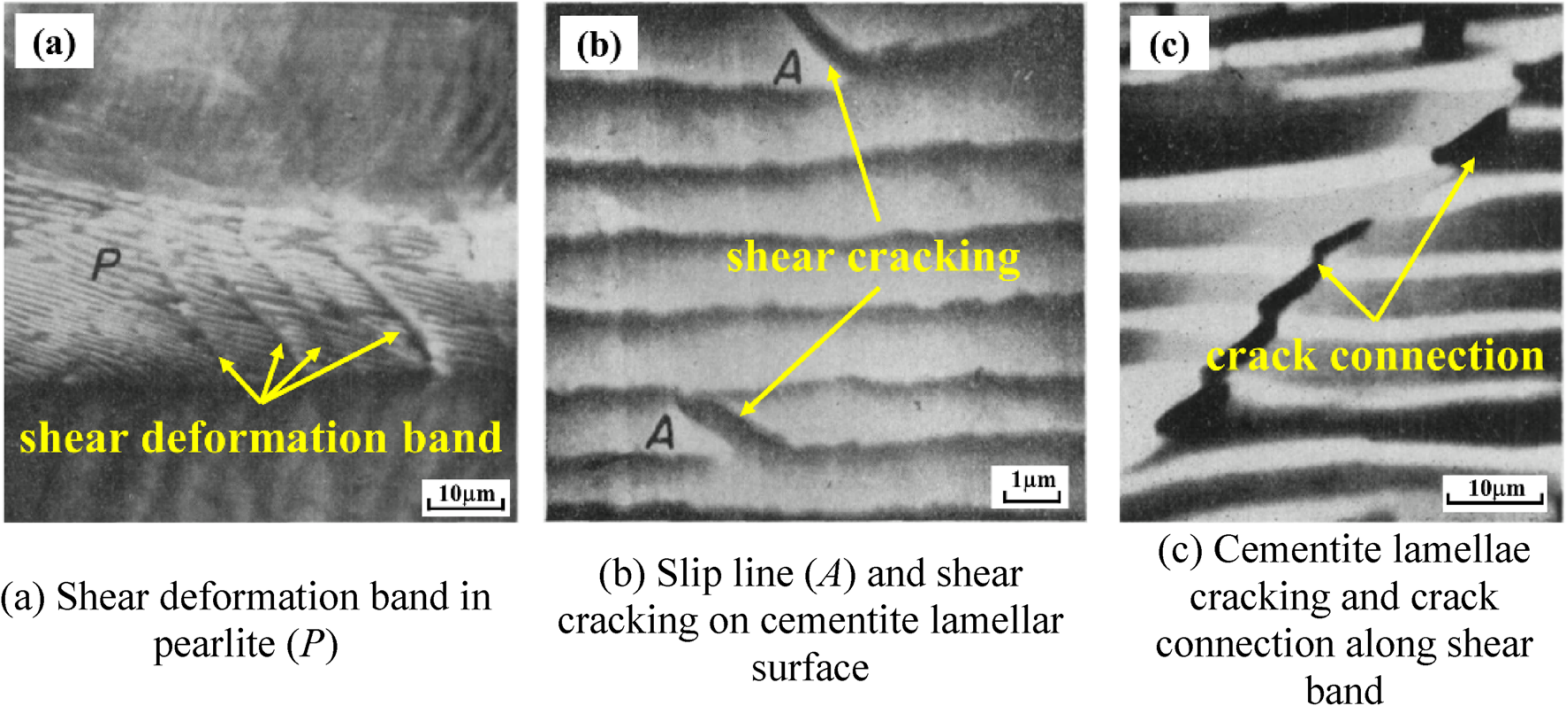
Deformation and crack initiation in pearlite^19^.

In the past few decades, many scholars have carried out a lot of research on ferrite–pearlite steel. Early scholars mainly focused on the deformation and fracture characteristics of layered pearlite steel from the macro scale^3–5^, revealing that the plastic deformation capacity of cementite lamella has a significant size effect, that is, when the thickness decreases to about 0.01 μm, it shows full plasticity^6,7^, and when the thickness increases to 0.1 μm, it shows full brittleness^8,9^. Relevant studies have also determined the quantitative compliance relationship between material strength and microstructure size. For example, ferrite strength is inversely proportional to the line intercept of structure size^10^, while pearlite strength is inversely proportional to the square root of its average interlayer spacing^11^. For the fracture mechanism of ferrite–pearlite steel, early studies showed that cracks could be initiated at the staggered wall when α-Fe deformation was large^12,13^. It is also considered that the slip of ferrite layer is blocked by cementite lamella and other lattice defects, resulting in stress concentration and crack initiation. Therefore, the crack can originate from the interior of pearlite crystal cluster^14^, and the initial crack of pearlite comes from cementite sheet, followed by ferrite fracture^15–18^. It is observed that the deformation in pearlite structure mainly occurs in the form of shear deformation as shown in Fig. 2a. In fine lamellar pearlite, cracks usually nucleate at inclusion interface and two-phase interface^19^ (Fig. 2b), which is consistent with the research results of crack nucleation at twin interface and grain boundary interface^20^, while coarse pearlite structure cracks along shear band (Fig. 2c), dislocations originating from the ferrite layer, and the double plug group of dislocations under the action of external stress^4^, under the combined action of normal stress and shear stress, lead to the fracture of cementite layer in the direction perpendicular to the tensile axis^19^. Some scholars have tried to explain the whole process of tensile deformation and crack initiation of ferrite–pearlite steel by means of experimental loading and dislocation theory^21^.

With the development of experimental observation means and continuum damage mechanics, scholars are more concerned about the effects of the characteristics of ferrite–pearlite steel microstructure components, mutual interaction, microstructure geometric characteristics, as well as their changes on the macro response of materials^22^. Because the damage and failure of materials involve multi-scale processes from micro to macro and the coupling between various levels, it is necessary to develop a multi-level meso analysis method. Previous studies have carried out systematic macro /meso mechanical comprehensive analysis on pearlite steel with layered meso structure, and given relevant numerical results. In particular, they have made a theoretical explanation for the phenomenon that brittle fracture is not observed in cementite layer with thickness less than 0.01 μm found in existing tests^6,7^, and also put forward the analysis principle of cementite thickness on crack initiation and evolution^23^.

Molecular dynamics (MD) provides an important way to understand the deformation and failure of metal materials. Decelis and Argon et al.^24^ studied the fracture behavior of brittle plastic transition at the top crack of α-Fe by molecular dynamics method, and the simulation results are in good agreement with the predicted results of Griffith theory and the experimental values. Professor Rice^25^ from Harvard University proposed the solid physical parameter of dislocation emission unstable stacking energy to describe the dislocation emission at the crack tip, instead of the concept of dislocation core in the simulation of crack initiation and propagation. Mullins^26^ simulated the brittle fracture propagation of the material and considered that the dislocation emitted by the crystal at the crack tip led to the passivation of the crack. Panin et al.^27^ deals with the impact deformation and fracture behaviour of commercial plain carbon pipe steel by theoretical method of excitable cellular automata and laboratory impact bending tests. Cao and Wang et al.^28^ simulated the deformation process of crack in uniaxial tensile experiment of α-Fe by molecular dynamics method, the deformation characteristics and fracture mechanism of cracks with different crystal orientations were studied, and the phenomena such as dislocation nucleation and emission, dislocation movement, stacking fault or twin formation, nano cavity formation and connection were observed. For the ferrite (α-Fe) and cementite (Fe_3_C) lamellae concerned in this paper. Mendelev^29^ proposed the FS-EAM potential function and its parameters of Fe. J. Hepburn and G.J. Ackland^30^ obtained the EAM/FS potential function of Fe–C alloy through first principle calculation, which was then improved by meam potential function^31^. Relevant research was obtained such as α-Fe/Fe_3_C interface structure^32^, cementite layer thickness effect^33^, deformation and failure mechanism^34^, etc. by MD technology. In addition, our research group also carried out relevant research on molecular dynamics simulation of pipeline steel microstructure in the past few years^35,36^.

As a new branch in the field of molecular scale, the serial cross scale method of cohesive zone model (CZM), which simulates and obtains traction separation (TS) relationship curve at atomic scale, endows the physical meaning and micro mechanism of failure parameters of CZM at atomic scale. Among the representative research results, Spearot et al.^37^ obtained the constitutive model of interface peeling of Cu single crystal boundary cracking through the molecular dynamics simulation, but it was not transformed into the TS relationship of local fracture surface. Yamakov et al.^38^ simulated the intergranular fracture of Al metal, qualitatively extracted the TS relationship curve based on molecular dynamics results, and did not give the accurate expression of TS curve. In addition, Komanduri et al.^39^ also simulated the deformation failure behavior of single crystals such as Ni, Fe and Cr. Komanduri only simulates uniaxial loading, while Spearot simulates combined loading of tension and shear. For multiphase materials, Awasthi et al.^40^ simulated the interfacial behavior of polyethylene and carbon nanotubes under tensile load. Zhou et al.^41,42^ also studied the interface failure behavior of different materials, focused on simulating the interface failure process of two kinds of BCC single crystals under the combined load of tension and shear, and obtained its TS relationship curve. Chinmaya et al.^43^ simulated the forward and tangential loading of Al-SiC composite system by molecular dynamics, imported the extracted TS curve into the finite element model as the interface failure criterion, obtained the stress–strain response of metal matrix composites at high strain rate, and compared it with the test results. In addition, using the CZM method based on molecular dynamics, Chinmaya et al.^44^ also analyzed the porosity effect of Al_2_O_3_-Al composites, and characterized the cohesion strength of TS curve by Weibull statistical distribution.

In the 1960s, ferritic-pearlitic steel was widely used in oil and gas pipelines, such as X52, X56, X60, X70 and other API steel grades. There is still a lack of research on the interface cracking of ferrite–pearlite steel, especially the interface cracking with Bagaryatskii crystal orientation relationship. Although Guziewski is specific to the α-Fe/Fe_3_C with Bagaryatskii crystal orientation relationship such as interface energy and interface dislocation have been studied, but the stable crack growth behavior of the configuration interface under external load needs to be further studied. Based on this, the micro degree of freedom of ferrite/cementite interface is analyzed in the first part of this study to judge the terminal type of crystal at the interface; Then, in the second part, by selecting the appropriate multi-body potential function, the ferrite/cementite molecular dynamics overlapping lamellar structure with initial defects is established, and the energy minimization based on conjugate gradient method (CG) and temperature annealing relaxation of canonical ensemble at constant temperature and pressure (NPT) are implemented to obtain a stable simulation system; In the third part, the crack initiation and development behavior under typical interface terminal types are analyzed, and the traction separation curves of cohesive zone with three thicknesses are extracted; Then, in the fourth part, the MD-CZM serial multiscale method is used to extract the reaction force displacement response curve based on the triangular cohesion model by embedding the cohesive element in the standard compact tensile (CT) specimen, and the fracture toughness of ferrite/cementite lamella under different interface terminal types is obtained; Finally, based on the above research results, the paper obtains several research conclusions.

## Characteristics of ferrite/cementite lamellar interface

### Grain orientation relationship

The arrangement of pearlite clusters and the orientation relationships (ORs) between ferrite and cementite determine the mechanical properties of ferrite–pearlite steel. Although scholars have done a lot of research on the microstructure and macro mechanical properties of pearlite, there are still many problems to be solved, especially at the interface of different phases, as one of the most vulnerable regions of materials, the research on the relationship between ferrite and cementite grains or in pearlite is particularly important. In earlier studies, transmission electron microscope (TEM) was used to study the ORs of pearlite. It was found that there are mainly the following three ORs in pearlite. Under the fixed definition of crystal direction, the crystal direction relationship is expressed as follows:

Bagaryatskii ORs:

1 $$\begin{gathered} \left[ {100} \right]_{\theta } \;\;\parallel \;\;\left[ {1\overline{1} 0} \right]_{\alpha } \hfill \\ \left[ {010} \right]_{\theta } \;\;\parallel \;\;\left[ {111} \right]_{\alpha } \hfill \\ \left( {001} \right)_{\theta } \;\;\parallel \;\;\left( {11\overline{2} } \right)_{\alpha } \hfill \\ \end{gathered}$$

Isaichev ORs:

2 $$\begin{gathered} \left[ {010} \right]_{\theta } \;\;\parallel \;\;\left[ {111} \right]_{\alpha } \hfill \\ \left( {103} \right)_{\theta } \;\;\parallel \;\;\left( {01\overline{1} } \right)_{\alpha } \hfill \\ \end{gathered}$$

Pitsch-Petch ORs:

3 $$\begin{gathered} \left[ {010} \right]_{\theta } 2.6^\circ {\text{from}}\left[ {131} \right]_{\alpha } \hfill \\ \left( {001} \right)_{\theta } \;\;\parallel \;\;\left( {\overline{2} \overline{1} 5} \right)_{\alpha } \hfill \\ \end{gathered}$$

where, *θ* and *α* represents cementite and ferrite respectively. Recently, the more advanced electron backscatter diffraction (EBSD) technology has been used to observe the microstructure of pearlite. The research has confirmed the discovery of TEM, but there is no consistent conclusion in the scientific community which of the three ors is the most common in pearlite. In addition, whether TEM or EBSD, these experimental techniques still stay in the macro ors expression on the scale, and do not deeply explore their atomic structure. In the study of ferrite/cementite interface, guziewski^32^ pointed out that bagaryatskii ors crystals are most likely to form interface structure in pearlite. Considering the influence of terminal plane chemical properties at the interface, guziewski studied the interface properties of bagaryatskii ors. However, the relevant studies are preliminary exploration without external load. In this study, pearlite with bagaryatskii ors is also selected to further study the crack propagation at the ferrite/cementite interface.

### Interface terminal type

**Figure 3:**
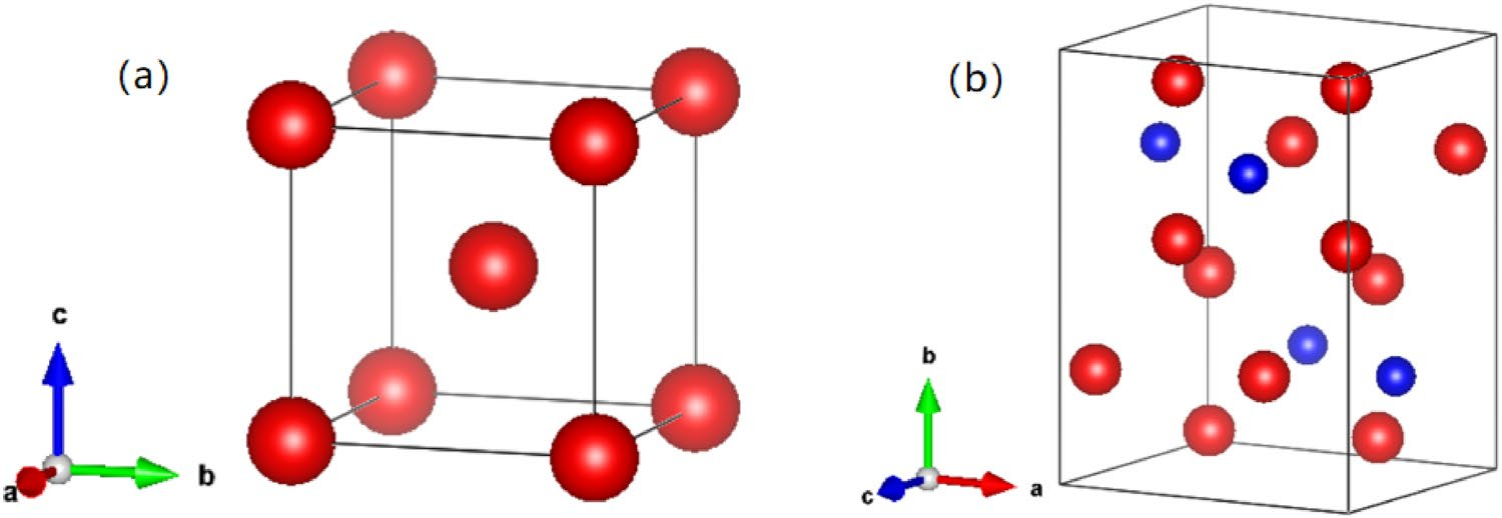
Crystal structure of ferrite (**a**) and cementite (**b**) (red indicates Fe and blue indicates C).

In the pearlite overlapping lamellar structure, ferrite has a typical body centered cubic structure, while cementite has a complex orthorhombic crystal structure, as shown in Fig. 3a–c represent three crystal directions [100], [010] and [001], respectively. The lattice constants of ferrite and cementite are shown in Table 1.

**Table 1:** Lattice constants of ferrite and cementite.

	a (Å)	b (Å)	c (Å)	α	β	γ
α-Fe	2.8664	2.8664	2.8664	90°	90°	90°
Fe_3_C	5.06	6.74	4.51	90°	90°	90°

However, in order to facilitate the establishment of the interface model of Bagaryatskii OR, it is necessary to reconstruct a single ferrite cell, that is, the ferrite cells arranged in a = [100], b = [010] and c = [001] are transformed into a =[1-10], b = [111] and c= [11-2] by coordinate transformation.

**Figure 4:**
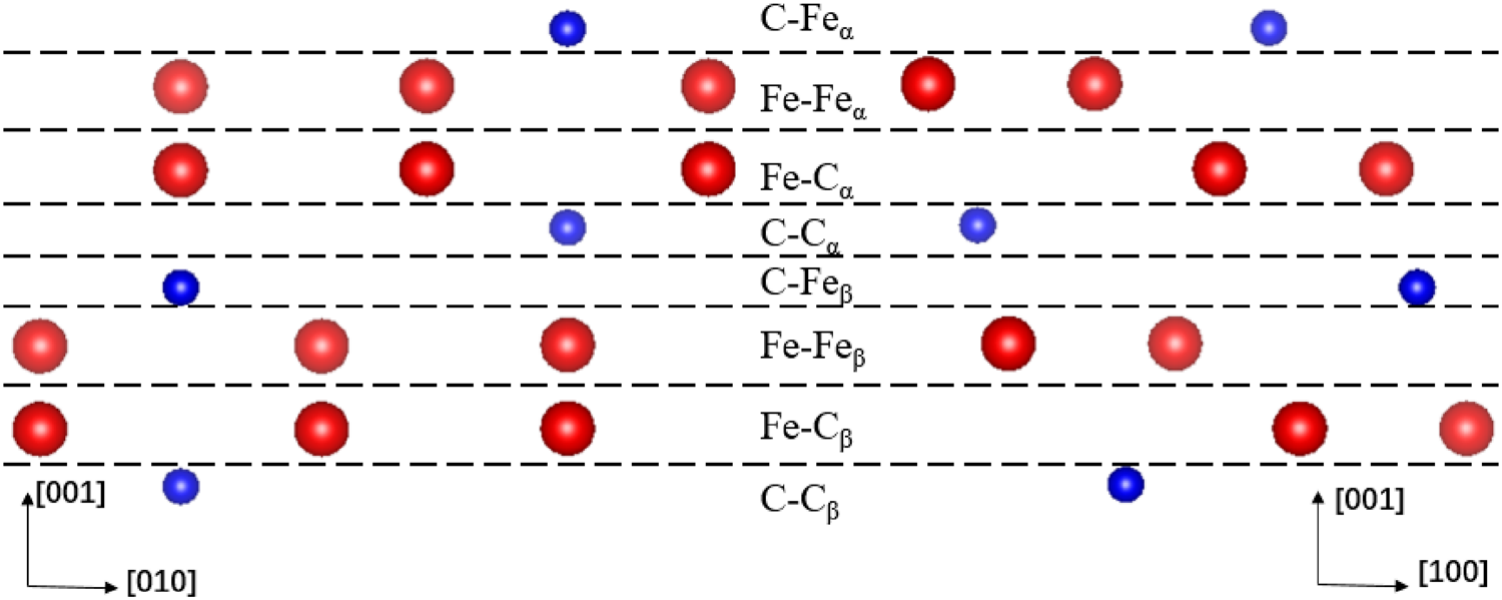
Plane classification of cementite terminal.

The properties at the ferrite/cementite interface are not only related to the crystal structure of ferrite and cementite, but also need to consider the influence of micro degree of freedom at the interface^32^. Because of the complex orthorhombic crystal structure of cementite, there are different configurations of termination planes at the (001) crystal plane and ferrite interface. Figure 4 shows the atomic structure of (100) and (010) crystal planes of cementite. Eight different cementite termination planes can be formed by cutting along (001) crystal plane at the dotted line. According to the difference of the outermost two atoms of cementite, the termination plane is defined as C–Fe_α_ plane, Fe–Fe_α_ plane, Fe–C_α_ plane, C–C_α_ plana, C–Fe_β_ plane, Fe–Fe_β_ plane, Fe–C_β_ plane and C–C_β_ plane. In the process of forming an overall interface with ferrite, because ferrite has symmetry in the <111> direction, if it is rotated 180 degrees, the terminal configuration of α plane and β plane is consistent. Therefore, the terminal types of cementite at the interface can be simplified into four types: C–Fe, Fe–Fe, Fe–C and C–C.

Secondly, the relative position of the two crystal termination planes is also an important factor affecting the micro degree of freedom of the interface. During the simulation, the relative position of the crystal plane is determined by moving the ferrite part. The initial distance between ferrite (111) crystal plane and cementite (001) crystal plane has not been studied in relevant literature. Zhang et al.^45^ found that the interface energy of Al_2_O_3_ and Al was stable near 2 Å, so the distance between the two crystal terminal planes is taken as 2 Å in this paper. At the same time, the ferrite also moves a certain distance randomly in the other two directions to ensure the stability of the model.

**Figure 5:**
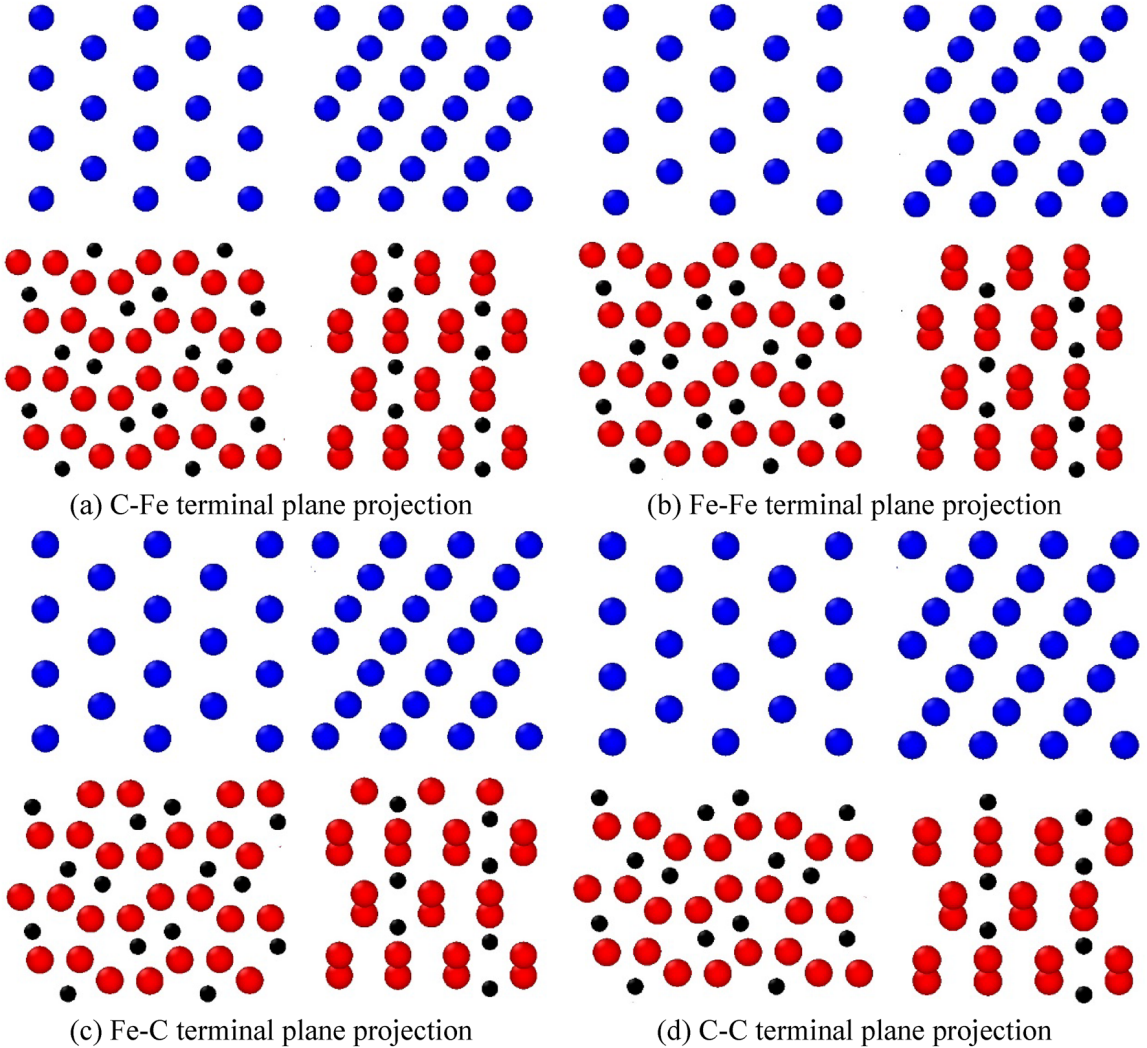
Projection of local structure at ferrite/cementite interface.

Figure 5 shows the local structure projection of the atomic model interface on the $\left( {010} \right)_{\theta } \;\;\parallel \;\;\left( {111} \right)_{\alpha }$ (left) and $\left( {100} \right)_{\theta } \;\;\parallel \;\;\left( {1\overline{1} 0} \right)_{\alpha }$ (right) crystal planes respectively. The initial distance between the lowest atom of ferrite crystal and the uppermost atom of cementite is always 2 Å. The details of the atomic model are given in Table 2. It can be seen from the table that the lattice mismatch in each direction is much less than 5%^46^, which meets the condition of complete coherent lattice.

**Table 2:** Details of atomic model.

Type	[100]_θ_ || [1-10]_α_		[010]_θ_ || [111]_α_		Height (Å)	Atoms
	Length (Å)	ε_x_ (%)	Length (Å)	ε_y_ (%)		
C–Fe	470.580	0.030	26.960	1.290	353.150	423,528
Fe–Fe					352.685	423,156
Fe–C					351.923	422,040
C–C					351.437	420,924

## Modeling and relaxation process

### Establishment of ferrite/cementite lamellar model

Based on the analysis of ferrite/cementite interface structure, a three-dimensional atomic model with central through crack is further established. Before modeling, the size of the model system needs to be designed because the system is too small. Due to the influence of size effect, the simulation will be inaccurate, and the system is too large, and the calculation time and cost are too long. The size determination of the model system is mainly based on two factors: one is the influence of lattice mismatch at the interface, and the other is the determination of the minimum simulation thickness. Lattice mismatch is a parameter to describe the lattice matching degree of substrate and epitaxial film. Lattice mismatch and coefficient of thermal expansion mismatch affect the epitaxial growth of crystals to varying degrees, resulting in a large number of defects in the epitaxial layer, or even the inability to grow single crystals, affecting the macro performance of components. Lattice mismatch can be approximately defined as^46^:

4 $$\varepsilon { = }2\left| {\frac{{a_{1} - a_{2} }}{{a_{1} + a_{2} }}} \right|$$

The minimum thickness affects the accuracy of local physical parameters. It has been proved that the local effect can be ignored when the model thickness is not less than 4 times the lattice constant^47^.

**Figure 6:**
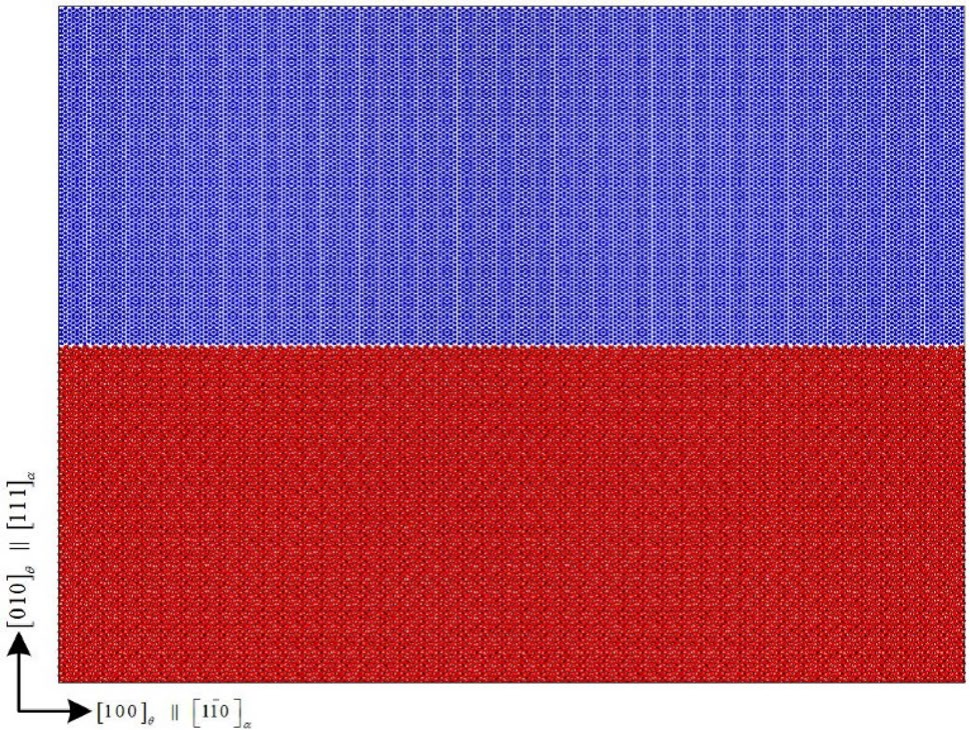
Ferrite/cementite interface model.

Based on the above principles, three-dimensional atomic models of four types of terminal interfaces are established in this work. Firstly, a partial cementite model is established, which contains 93, 4 and 39 single cells in three directions, with a size of 47.058 nm × 2.696 nm × 17.589 nm, containing 232,128 atoms, as shown in the blue part of Fig. 6. Then the ferrite partial model is established, which contains 116, 11 and 25 single cells in three directions, with a size of 47.023 nm × 2.731 nm × 17.553 nm, containing 191,400 atoms, as shown in the red part of Fig. 6. Finally, the two models are combined along the Z direction to construct the ferrite/cementite interface model. Finally, the relative position of the plane is determined by moving some ferrite crystals, and the atomic models with different termination plane structures are established by deleting the outermost atom of the ferrite crystal at the interface.

### Selection of potential function

Four typical potential functions of iron carbon system are selected for comparison in this paper, including Lennard Jones/Morse potential function^48–50^, EAM potential function^51^, Tersoff potential function^52^ and MEAM potential function^53^. The parameters of Lennard Jones/Morse potential function are given in Table 3, and the calculation results of elastic constants of ferrite and cementite single crystals are given in Tables 4 and 5 respectively. From the calculation results of ferrite elastic constants, with the exception of Lennard Jones/Morse potential function, the calculation results of the other three potential functions are not different from the experimental results. From the cementite calculation results, only the calculation results of elastic constants of Tersoff and MEAM potential functions are in good agreement with the first principle calculation results. MEAM potential function has higher accuracy, but the calculation cost is high. Therefore, considering both accuracy and cost, Tersoff potential function is selected in the subsequent simulation process.

**Table 3:** Parameters of Lennard Jones/Morse potential function.

Morse potential	D (eV)	*α* (Å^−1^)	*r*_0_ (Å)
Fe–Fe	0.417	1.389	2.845
Fe–C	0.153	1.268	2.219

Lennard–Jones potential	*ε* (eV)	*σ* (Å)	
C–C	0.003	3.468

**Table 4:** Calculation results of ferrite elastic constants.

	Lennard–Jones/Morse	EAM	Tersoff	MEAM	Experimental
C_11_ (GPa)	183.425	243.685	219.384	211.825	242.000
C_12_ (GPa)	150.186	145.005	142.842	142.388	147.000
C_44_ (GPa)	149.968	115.931	130.245	117.883	112.000

**Table 5:** Calculation results of cementite elastic constants.

	Lennard–Jones/Morse	EAM	Tersoff	MEAM	DFT
C_11_ (GPa)	301.179	251.351	387.520	324.008	388.000
C_22_ (GPa)	324.669	247.487	356.562	230.965	345.000
C_33_ (GPa)	295.786	331.846	419.602	332.414	322.000
C_44_ (GPa)	82.468	57.294	50.339	17.934	15.000
C_55_ (GPa)	84.481	157.826	133.488	102.860	134.000
C_66_ (GPa)	55.298	63.018	131.644	92.162	134.000
C_12_ (GPa)	83.040	26.035	184.300	137.135	156.000
C_13_ (GPa)	106.264	171.030	181.593	163.802	164.000
C_23_ (GPa)	109.558	20.194	149.650	121.719	162.000

### Minimum energy sample acquisition

The initial configuration of the simulated sample at the grain boundary seriously affects the mechanical behavior of the cohesive zone simulation. Moreover, due to the limitation of time scale of molecular dynamics simulation, it is difficult to make the grain boundary atoms reach the lowest energy state (second order) in the experiment. Therefore, choosing an appropriate relaxation method to achieve the lowest energy state of the initial configuration of crystal samples is very important to obtain the real mechanical properties of polycrystalline metals. In the molecular dynamics simulation of nano polycrystalline metal samples carried out by Ma et al.^54^, the residual stress in the sample can be effectively reduced to the energy state of the test sample by two-step relaxation of static optimization of energy minimization and NPT dynamic optimization under constant temperature and pressure.

#### Energy minimization based on conjugate gradient (CG)

The process of energy minimization is actually the process of minimizing the objective function of Eq. (5). The objective function is the total potential energy of the system, which is a function of the coordinates of N atoms in the system, and its calculation formula is:

5 $$\begin{aligned} E\left( {r_{1} ,r_{2} , \cdots ,r_{N} } \right) &= \sum\limits_{i,j} {E_{pair} \left( {r_{i} ,r_{j} } \right)} + \sum\limits_{ij} {E_{bond} \left( {r_{i} ,r_{j} } \right) + \sum\limits_{ijk} {E_{angle} \left( {r_{i} ,r_{j} ,r_{k} } \right)} } + \hfill \\ \, & \sum\limits_{ijkl} {E_{dihedral} \left( {r_{i} ,r_{j} ,r_{k} ,r_{l} } \right)} + \sum\limits_{ijkl} {E_{improper} \left( {r_{i} ,r_{j} ,r_{k} ,r_{l} } \right)} \\ & \quad + \sum\limits_{i} {E_{fix} \left( {r_{i} } \right)} \hfill \\ \end{aligned}$$

The first term is the sum of all pair potential interactions without bond interaction, including long-range Coulomb interaction; The second to fifth terms represent the interaction of bond, angle, dihedral and improver, respectively; The last item considers the effects of constraints or forces on atoms.

#### Temperature rise annealing relaxation method in NPT ensemble

For polycrystalline samples, there will be large residual stress at the grain boundary after energy minimization, and the system is still far from the thermodynamic equilibrium state. Therefore, it is necessary to relax the system with global energy minimization. After the energy of the system is minimized by the CG method, the atomic kinetic energy is basically zero, which can not cross the potential barrier to reach the global minimum energy state, but can only stay in the local minimum energy state. In order to make the atoms move fully, find the lowest energy state of the system and eliminate the high stress still existing in the system after the first relaxation, the Parrinello-Rahman method and Nosé-Hoover method are used to control the stress and temperature respectively, that is, the temperature rise annealing relaxation under NPT ensemble is an effective method.

#### Relaxation effect analysis

**Figure 7:**
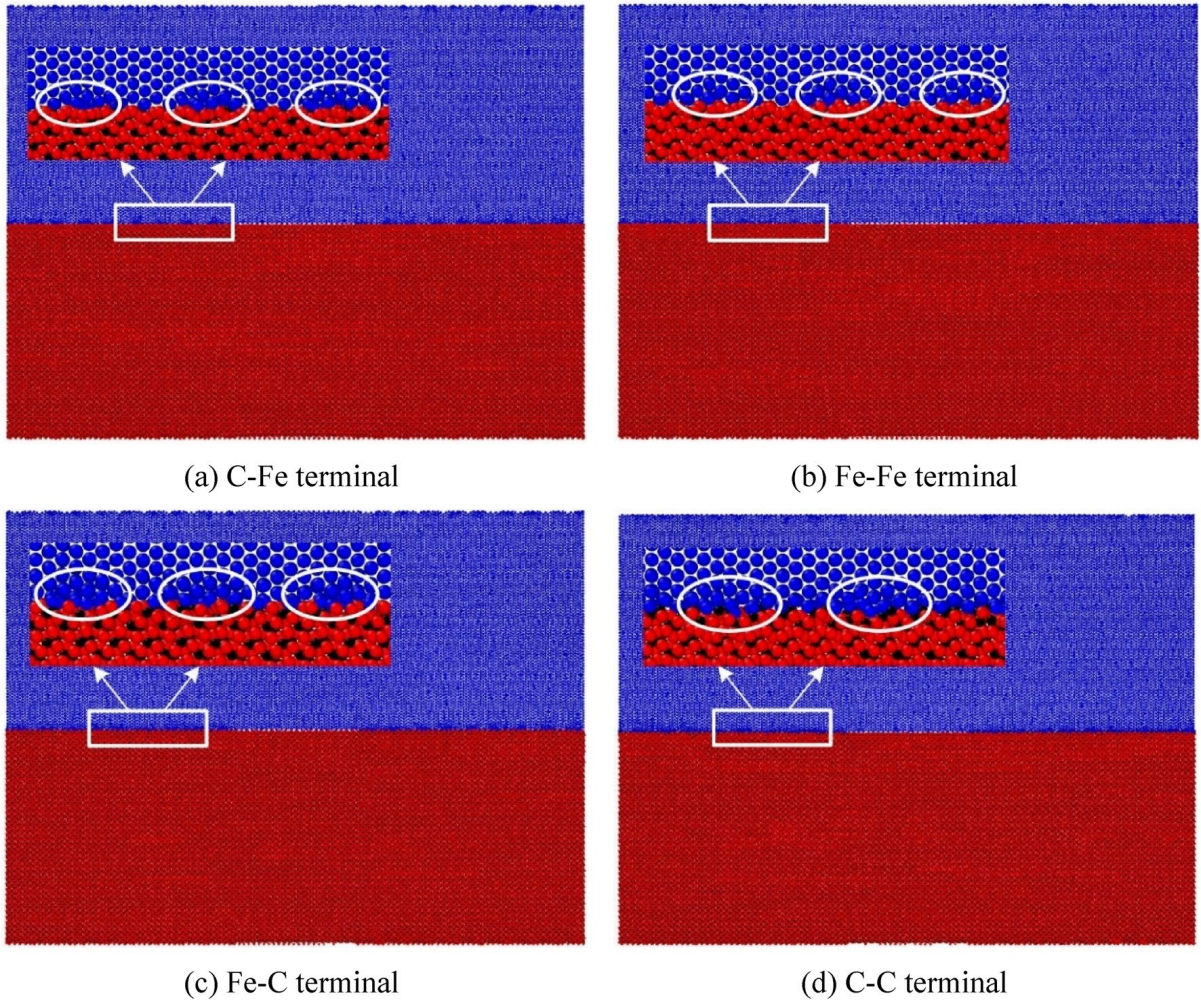
Sample morphology after relaxation.

**Figure 8:**
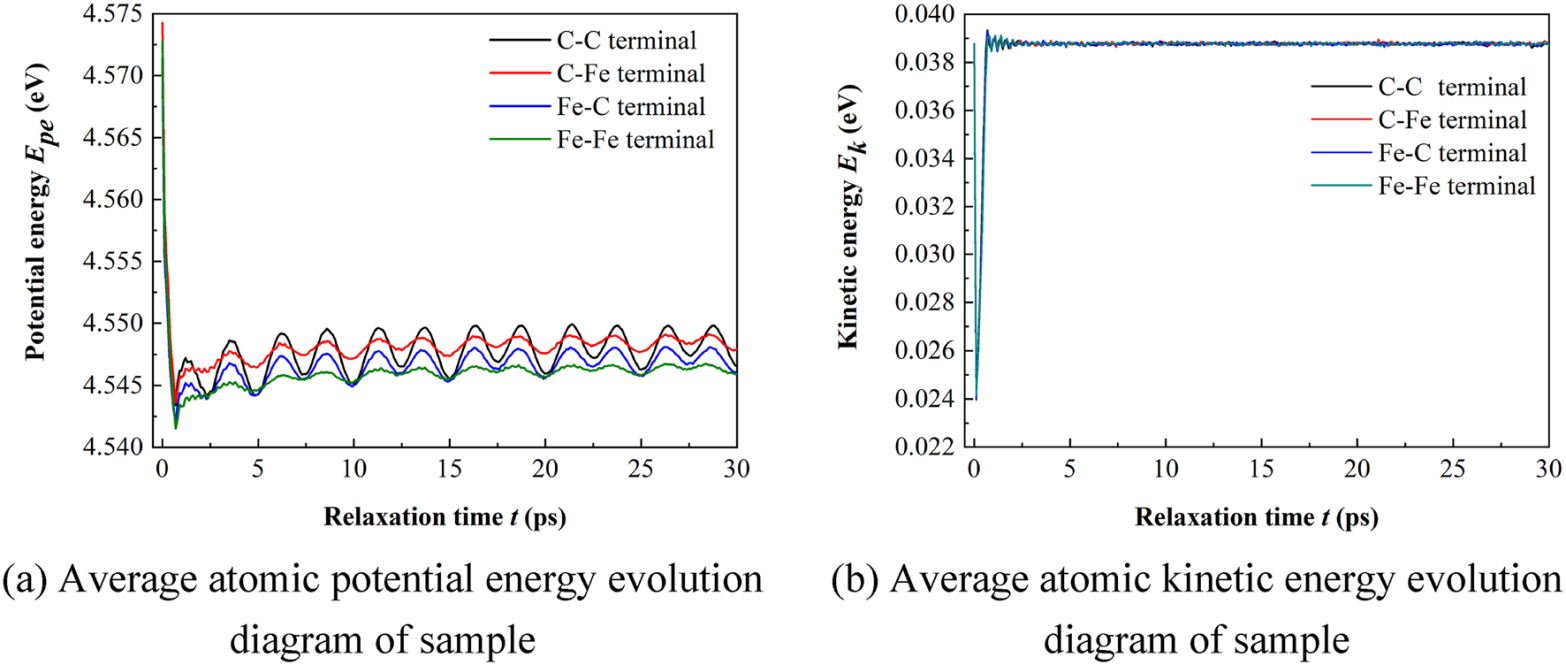
Evolution of energy with relaxation time sample.

**Figure 9:**
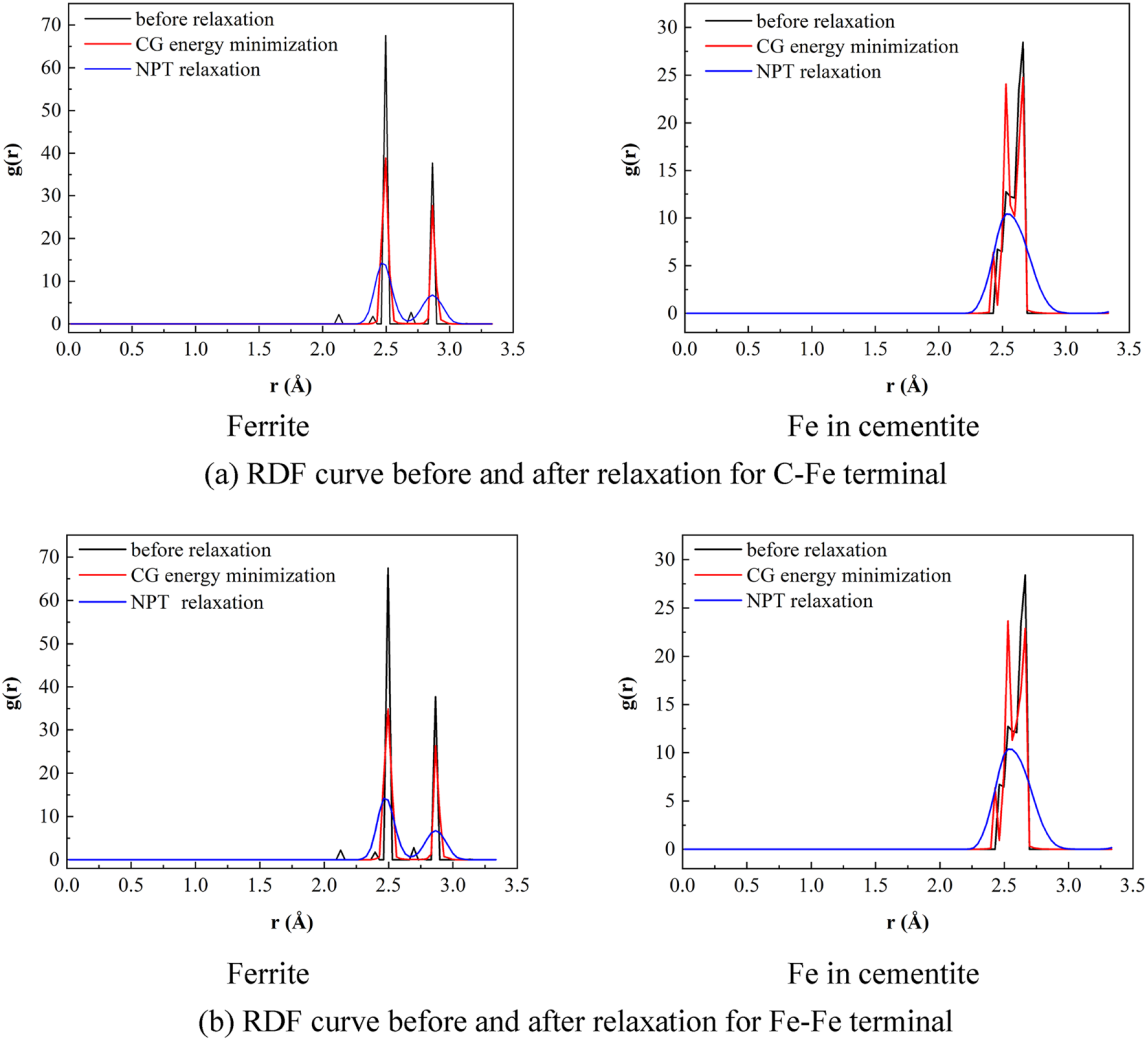

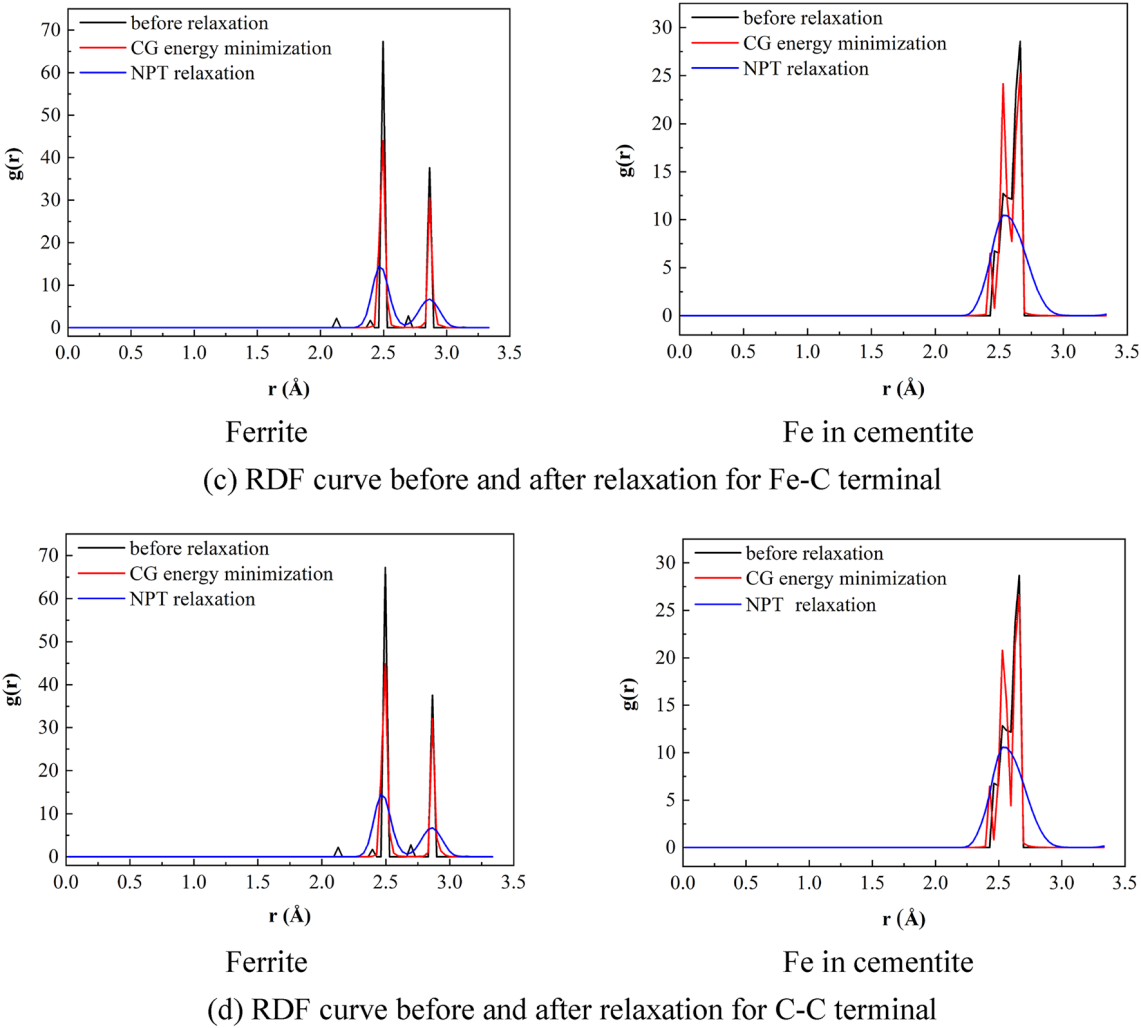
Radial distribution function curve before and after relaxation.

In this paper, the temperature is kept constant at 300 K during the simulation process. Periodic boundary conditions are used in all three directions. After minimizing the energy of the conjugate gradient method, the NPT relaxation at 300 K and 0 bar was carried out for 30 ps. After the relaxation, from the sample morphology, except for the prefabricated cracks at the grain boundary, the initial distance of 2 Å between the upper and lower interfaces no longer exists, and even some ferrite atoms and cementite are combined into the same grain as shown in Fig. 7 (at the white circle in the figures). After the sample average original potential energy changes sharply at the initial stage of relaxation, it gradually tends to be stable after 10 ps, while the sample average atomic kinetic energy quickly tends to be stable after sharp oscillation, as shown in Fig. 8. From the perspective of energy, the energy reaches equilibrium after CG energy minimization and 10 ps NPT relaxation. In order to compare the bonding law of ferrite and cementite before and after relaxation and the order of the two crystals, the radial distribution function (RDF) of Fe in ferrite and cementite before and after relaxation is calculated respectively, as shown in Fig. 9. It can be found that the radial distribution function before and after relaxation has two obvious characteristics: firstly, the maximum peak decreases in varying degrees, the adjacent peaks merge with each other, and the weak peaks disappear; Secondly, the radial distribution curve of Fe after relaxation still approaches 0 after the wave peak. The decrease and broadening of the maximum peak may be due to the atomic lattice mismatch at the interface after relaxation, which will lead to partial atomic rearrangement; The radial function value is still close to 0 after relaxation, indicating that the crystal continues to maintain a certain order.

On the whole, the crystal order is kept within an acceptable range, and the atomic nearest neighbor position changes little before and after two-step relaxation. Therefore, it is reasonable to minimize CG energy and 30 ps NPT relaxation.

## TS curve extraction of interlayer cracking process

### Definition of cohesive zone

The damage evolution of material fracture failure is based on the traction separation law of crack interface, and the crack path of material is defined by a CZM zone with zero thickness. Therefore, in atomic simulation, on the one hand, it is necessary to define the calculation method of traction and separation, and on the other hand, it is necessary to define CZM region. In the atomic model, the atomic region near the crack [i.e. grain boundary (GB)] in front of the crack tip is generally set as the cohesion region. For the discrete atomic model, the atomic stress is redefined in the atomic simulation. In this paper, the Virial formula is used to calculate the local stress^38^ *σ*_*αβ*_:

6 $$\begin{aligned} \sigma_{\alpha \beta } { = }\frac{1}{\Omega }\sum\limits_{i \in \Omega } {\left( {m^{\left( i \right)} v_{\alpha }^{\left( i \right)} v_{\beta }^{\left( i \right)} - \frac{1}{2}\sum\limits_{j} {\frac{\partial U}{{\partial r^{{\left( {i,j} \right)}} }}\frac{{r_{\alpha }^{{\left( {i.j} \right)}} r_{\beta }^{{\left( {i.j} \right)}} }}{{\left| {r^{{\left( {i,j} \right)}} } \right|}}} } \right)} \end{aligned}$$

where, *α*, *β* represents x, y and z directions, *m*^(*i*)^ and *v*^(*i*)^ represent the mass and velocity of atom *i* respectively, *U* is the atomic energy under different potential functions, and *r*^(*i*,*j*)^ is the displacement vector between atoms *i* and *j*. In the Eq. (6), the sum of the first term is obtained by calculating the local stress of all selected atoms, and the sum of the second term is obtained by calculating the interaction between atoms *i* within the truncation radius of atom *j*. The local volume Ω is theoretically the sum of the individual volumes of the atoms in the group. However, for some solid and liquid materials that are easy to deform, it is difficult to accurately define the individual atomic volume. Therefore, in LAMMPS, the atomic volume of a single atom is estimated by calculating its voroi volume, and the atomic volume of cohesion zone is obtained after summation.

**Figure 10:**
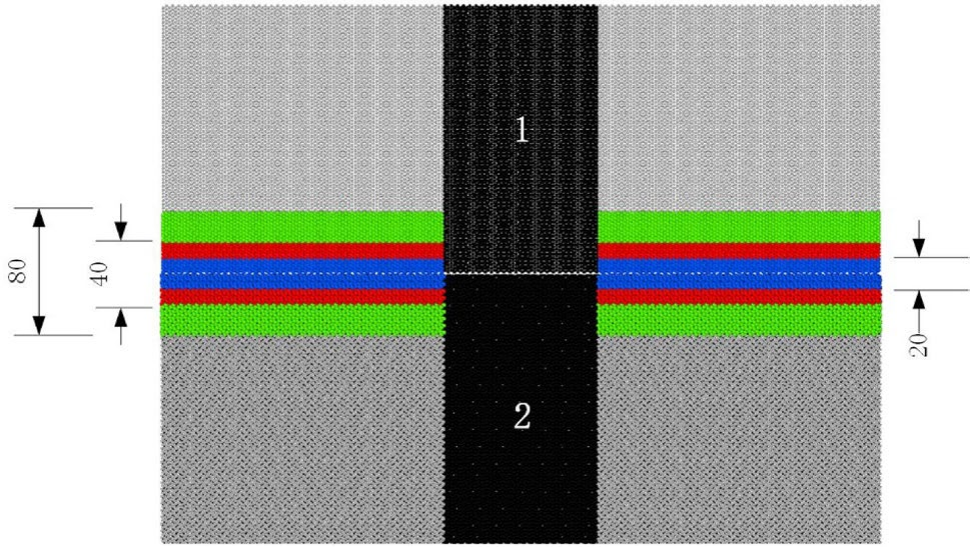
Definition of cohesive zone at atomic scale.

Since the simulated loading mode is uniaxial tension, the traction force in each area is expressed by the loading direction component of the total stress in the area, and the separation is also transformed from the loading direction component of the total strain. This is also a general practice of extracting TSL based on molecular dynamics^38^. Therefore, in the process of simulating type I cracking, the local stress in the cohesive zone can be *σ*_zz_ as the traction of the cracking interface. Because the cracked interface divides the atomic cohesion region into upper and lower parts, the separation of the interface can be obtained by the difference between the average displacements of atoms in the upper and lower cohesion regions. So far, the cohesion region and tension displacement criterion at the atomic scale have been defined. In order to analyze the thickness effect of cohesive zone, the TSLs of cohesive zone with thickness of 20 Å, 40 Å and 80 Å shown in Fig. 10 are compared and analyzed here.

Through further observation, it is found that there are phenomena such as atomic accumulation at the crack tip, vacancy formation at the trailing edge of the crack tip, hole growth and atomic bonding in the process of interface propagation. Figure 12 specifically reflects the dynamic process of C-Fe terminal type interface.

### Molecular dynamics simulation results

#### Stress–strain relationship

**Figure 11:**
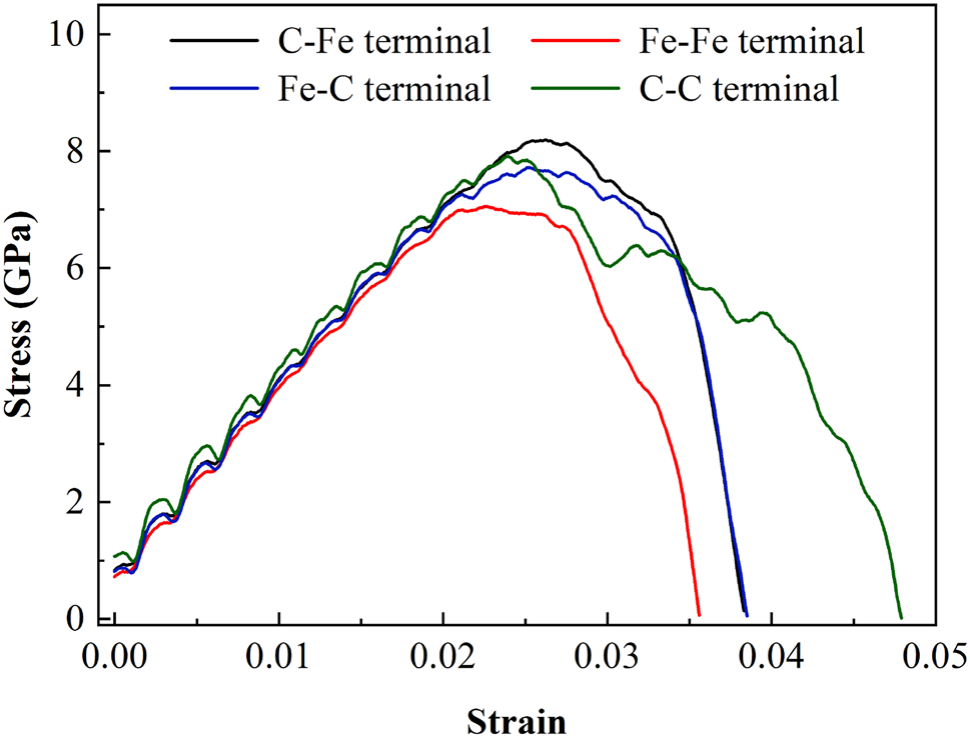
Stress–strain relationship of interface model during axial tension.

Figure 11 shows the stress–strain curves of four terminal interface models in the loading direction at a temperature of 300 K. In Table 6, mechanical parameters such as maximum tensile stress and corresponding strain are extracted from the stress–strain curve. It can be seen from the figure that under the condition of mode I fracture, the four terminal models have experienced an elastic process in which the stress increases linearly with the loading stress variable. The crack expands rapidly due to the fracture of inter atomic chemical bond after reaching their respective critical strain, the tensile stress gradually decreases from the maximum value to the blunting stage, and the material fails rapidly with the increase of crack growth rate, the stress is then reduced to zero. In the potential function test, it is known that the elastic modulus of ferrite in [112] direction is 242 GPa and that of cementite in [001] direction is 322 GPa. The elastic modulus of the four models is between 242GPa ~ 322GPa as shown in Table 6, and the maximum principal stress of C–Fe and C–C terminal is greater than that of Fe–Fe and Fe–C terminal, which may be that the interface is more stable when the carbon atom is located at the outermost layer of cementite terminal. At the same time, it should be noted that when the cementite interface terminal is the Fe–Fe terminal model in which the outermost two layers do not contain C atoms, not only the maximum principal stress is much less than that of other terminal models, but also the stress decreases to zero first to reach the failure state. When the cementite interface terminal is the outermost two layers are C atoms, The failure strain is also the maximum in the four terminals.

**Table 6:** Mechanical parameters during axial tension.

Terminal type	Maximum tensile stress *S* (GPa)	Strain at maximum stress *ε*	Fitted elastic modulus *E* (GPa)
C–Fe	8.188	0.026	296.380
Fe–Fe	7.052	0.023	300.630
Fe–C	7.718	0.025	293.830
C–C	7.912	0.024	300.790

#### Crack propagation process

**Figure 12:**
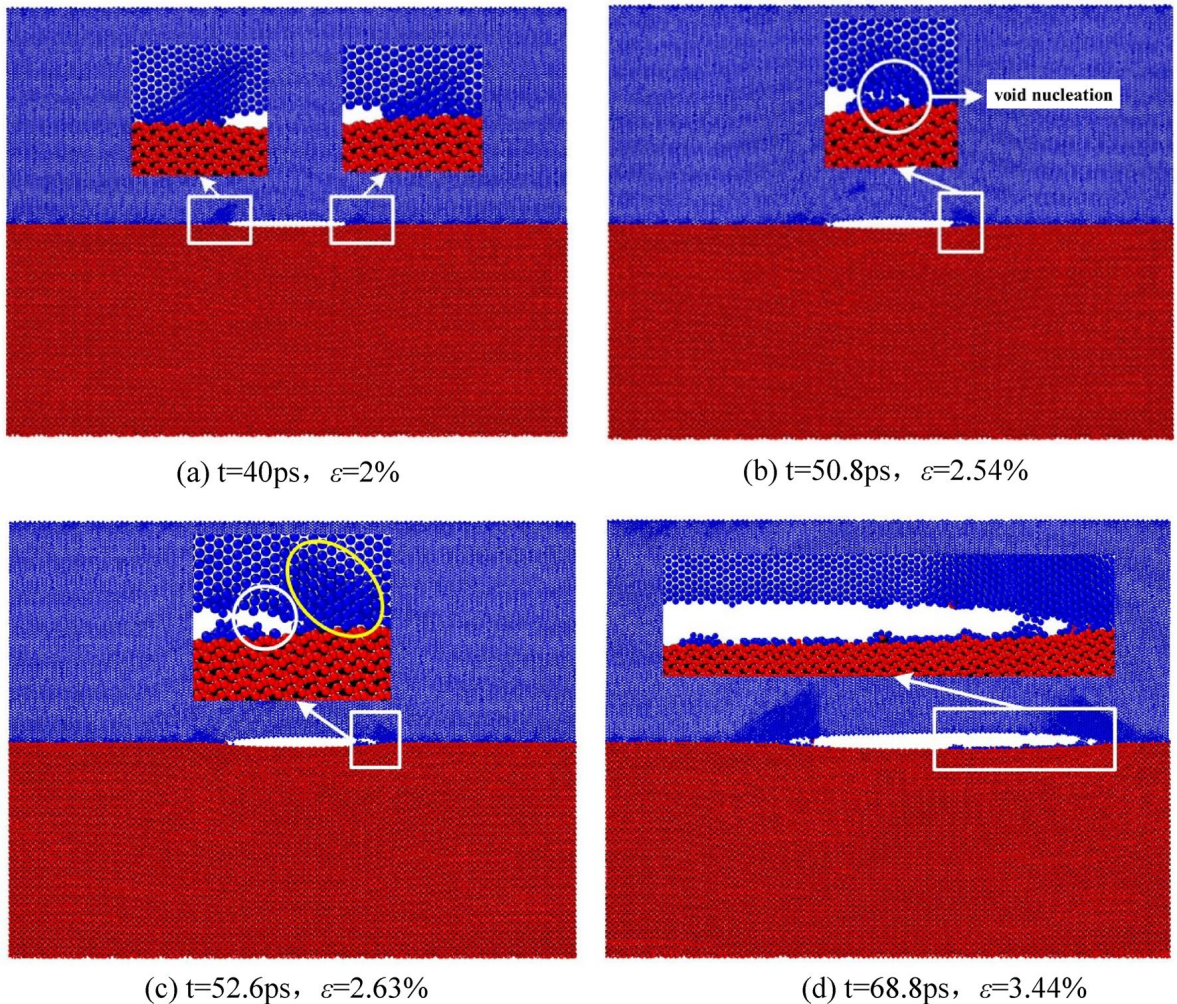
Atomic accumulation, hole initiation, hole growth and bonding of C–Fe terminal crack.

Figure 12A corresponds to the instantaneous model when the strain is 2% at 40 ps, the opening at the crack tip becomes larger with the gradual loading, the atoms at the crack tip rearrange and slowly deviate from the ideal lattice position and gradually accumulate at the crack tip. Compared with the initial configuration, the atoms at the crack tip are distorted and their order is destroyed, resulting in the plastic deformation at the crack tip. Figure 12b corresponds to the instantaneous model when the strain is 2.54% at 50.8 ps. With the further enhancement of plastic strain, the interaction between a few atoms decreases, and voids appear between atoms. With the expansion of voids, voids between atoms are formed. Figure 12c corresponds to the instantaneous model at 52.6 ps and strain of 2.63%. With the increase of interatomic vacancies, holes are gradually formed at the trailing edge of the crack tip (as shown in the white circle in Fig. 12c). With the growth and aggregation of holes, the upper and lower interfaces slowly separate, the interface cracks and forms a new crack tip, and the protons pile up again at the new crack tip (as shown in the yellow circle in Fig. 12c). After repeating the above process, the crack continues to grow forward. Figure 12d corresponds to the instantaneous model at 68.8 ps and 3.44% strain. It can be seen from the figure that in the process of interface separation, some Fe atoms (blue atoms) of ferrite crystals are attached to cementite. At the same time, some atoms of cementite (red Fe atoms and black C atoms) are separated upward with the ferrite part, and obvious bonding occurs between atoms.

### Thickness effect of cohesive zone

**Figure 13:**
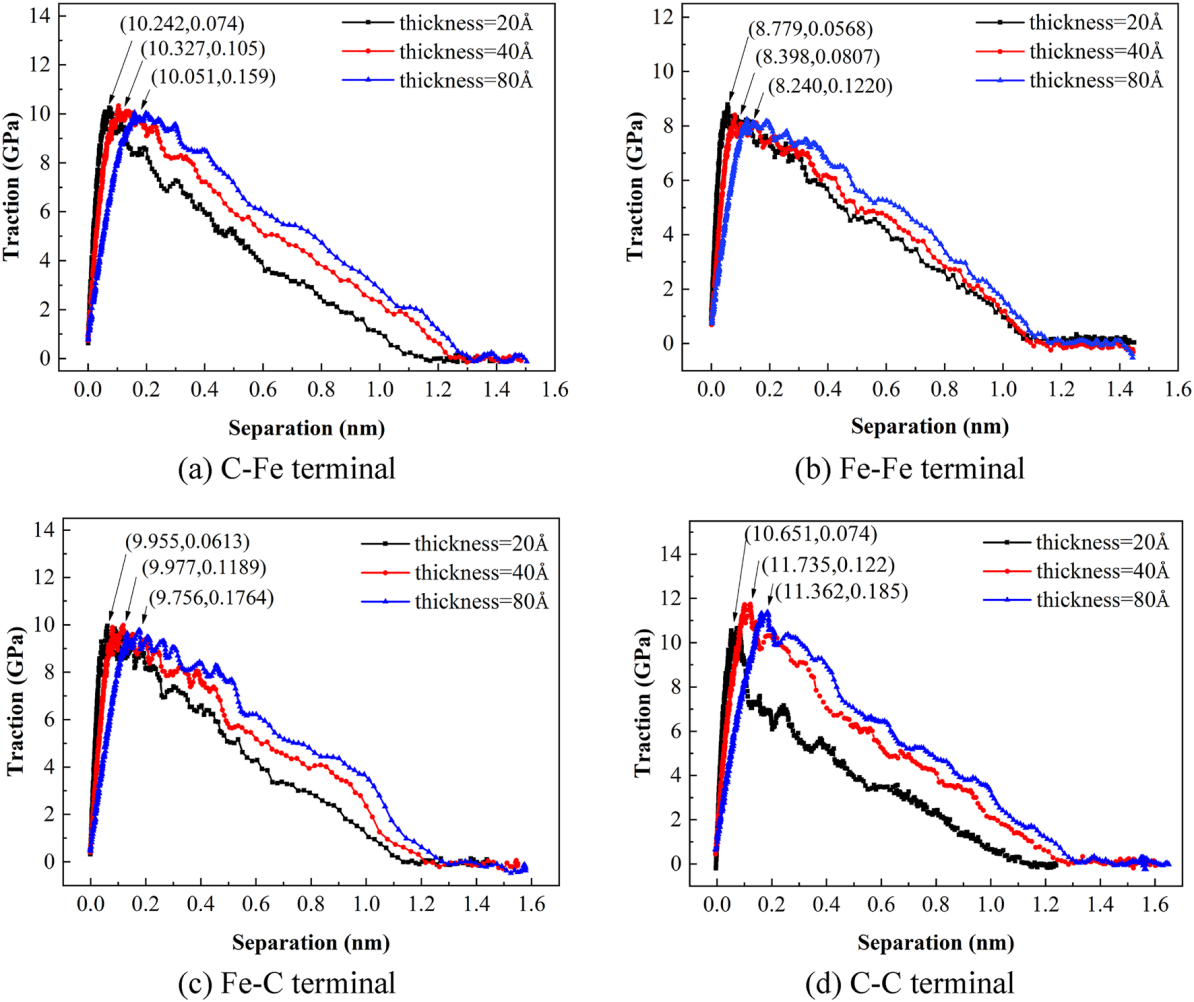
TS curves of different thickness cohesive zones with different terminals.

By analyzing the crack propagation at the ferrite/cementite interface at the atomic scale, the cohesion zone TS curves of the above three thicknesses are extracted, as shown in Fig. 13.

**Figure 14:**
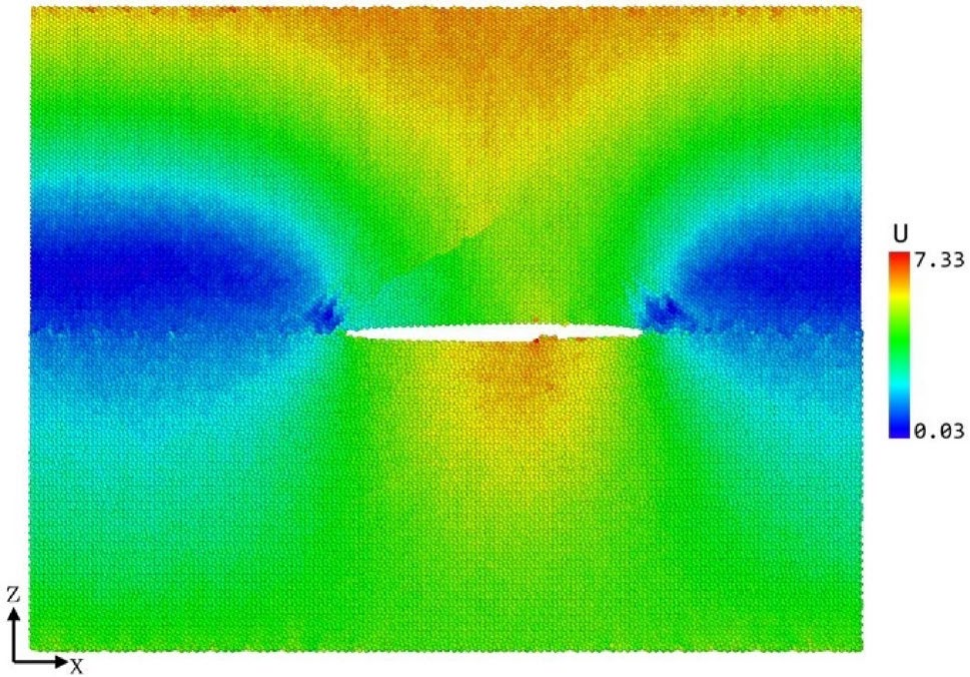
Cloud diagram of atomic displacement distribution at 60 ps of C–Fe terminal model.

As can be seen from Fig. 13, the maximum interface stress difference of the TS curve extracted with different thickness is not obvious. However, the separation displacement increases with the increase of thickness. Figure 14 shows the cloud diagram of atomic displacement distribution when the simulation time of C-Fe terminal model is 60 ps and the strain is 3%. Because the atoms far away from the crack do not have obvious plastic deformation, their displacement is much larger than that near the crack. With the increase of the thickness of the cohesive zone, the larger the proportion of the atomic displacement without plastic deformation to the calculated average displacement, resulting in the backward displacement of the characteristic displacement of the maximum stress peak at the interface. On the other hand, when the definition of the thickness of the cohesive zone is too small to include the whole plastic deformation zone, the blunting phenomenon in crack propagation cannot be explained. From the TS curve of 20 Å as shown in Fig. 13d, after reaching the stress peak, the crack tip expands rapidly and the stress decreases sharply Therefore, the definition of cohesive zone thickness is unreasonable, which cannot characterize the local propagation process near the crack, resulting in large errors in the calculated cohesive energy and other fracture parameters.

### Extraction of cohesive zone parameters

**Figure 15:**
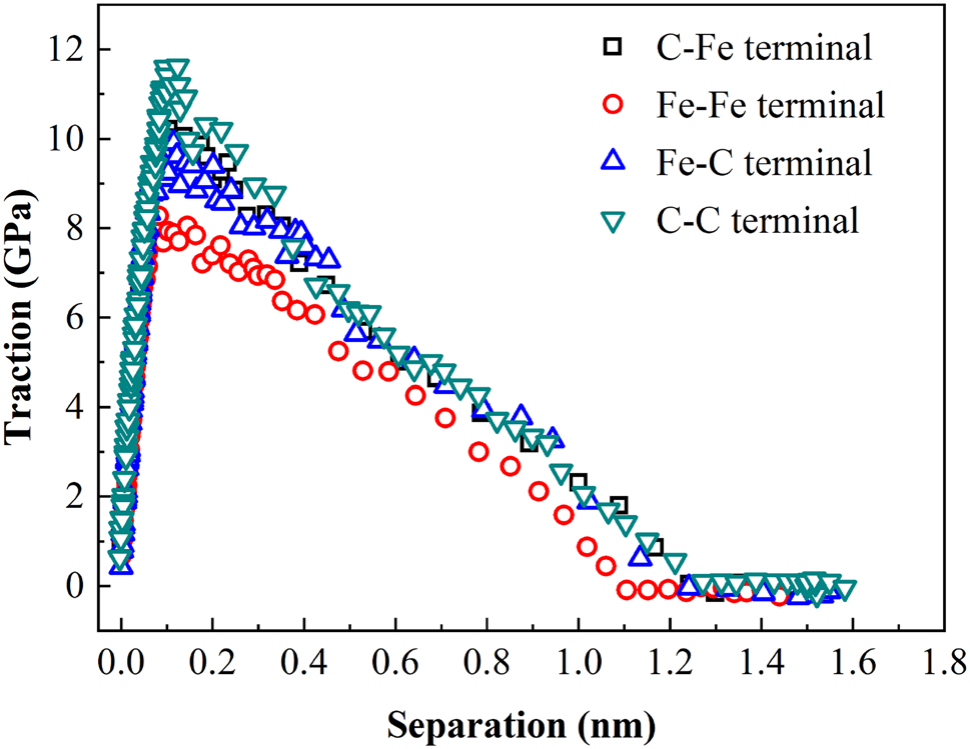
TS curve of four terminal models.

Based on the analysis above, the thickness of ferrite/cementite intergranular fracture cohesion zone is defined as 40 Å. Figure 15 shows the traction separation curves of intergranular fracture at the atomic scale of four terminal models. Table 7 shows the cohesion model parameters such as the extracted maximum stress at the interface, the separation displacement corresponding to the maximum stress and cohesion energy.

**Table 7:** Cohesion model parameters of four terminal models.

Terminal type	Maximum interfacial tension *T*_max_ (GPa)	Displacement at maximum tension *δ* (nm)	Cohesive energy *G*_C_ (N/m)
C–Fe	10.327	0.105	6.393
Fe–Fe	8.398	0.081	4.980
Fe–C	9.977	0.119	6.254
C–C	11.735	0.122	6.771

It can be seen from Table 7 that the cohesion energy of the four terminal interfaces is C–C > C–Fe > Fe–C > Fe–Fe from large to small, that is, the outermost layer of cementite terminal is carbon atoms, and the more the number of C atoms in the outermost two layers, the greater the cohesion energy, and more energy needs to be absorbed in the process of crack propagation. By extracting the parameters of the cohesive zone near the crack, the previous analysis results of stress and strain in the loading direction of the model are further explained, that is, C atom enhances the stability of the interface. This shows that the structure of cementite terminal interface has a crucial influence on the properties of two-phase interface.

## Description of crack propagation behavior of pipeline steel based onMD-CZM

### Establishment of finite element model

**Figure 16:**
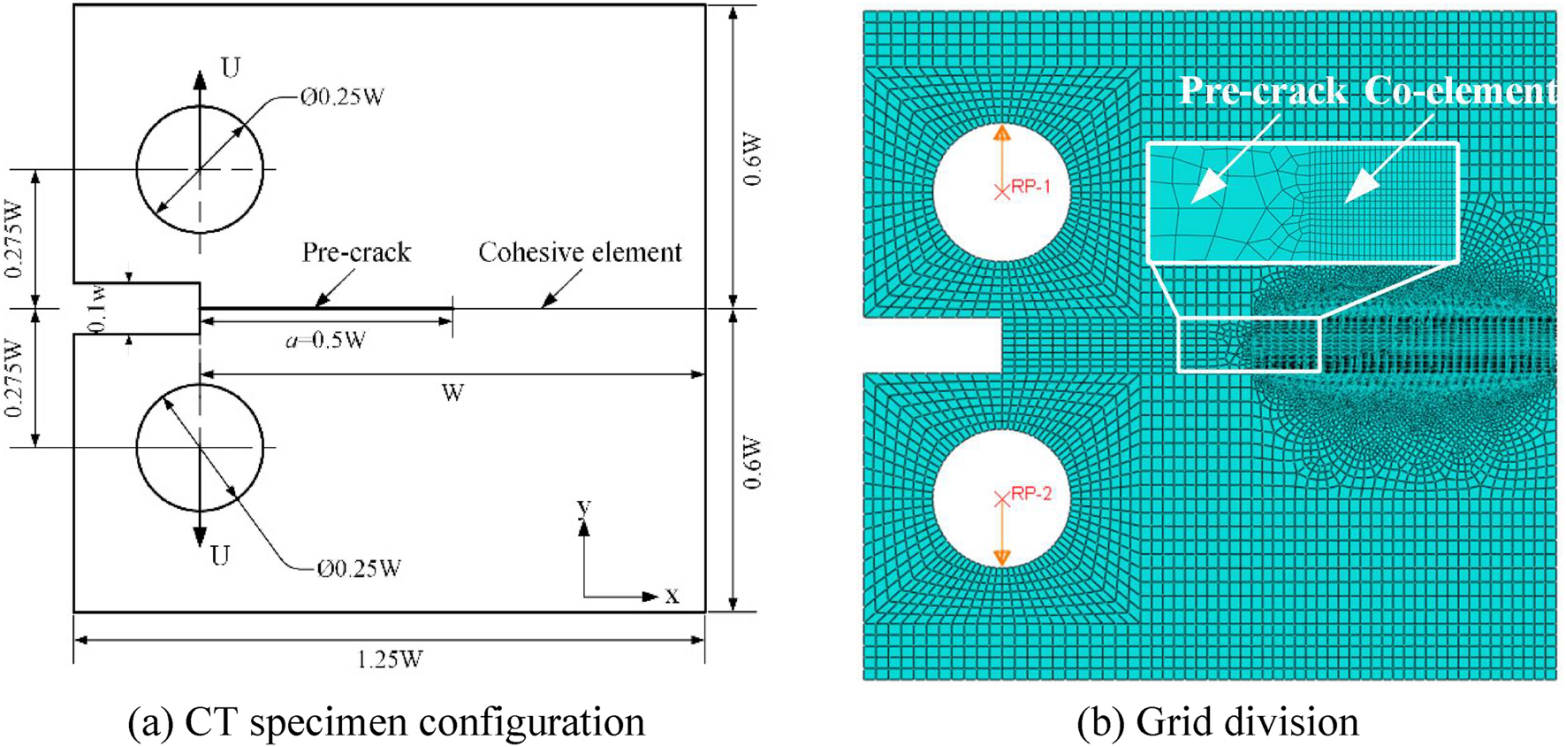
Schematic diagram of CT model.

A 2D plane strain compact tensile (CT) finite element model is established according to the ASTM standard size standard, and its size configuration can be found in Fig. 16a. The width of the model is W, the length of the prefabricated crack a = 0.5 W, the length of the cohesive unit area, i.e. the crack propagation area, is also 0.5 W, the notch width of the mechanical crack is 0.1 W, the diameter of the pin hole is 0.25 W, and the size of the whole model is 1.25 W × 1.2 W. The external load is applied by displacement loading. In order to facilitate loading, the contact part between the model and the pin is coupled to the reference points RP-1 and RP-2 respectively, and the displacement load u with opposite direction and equal size is applied. The cohesion area is meshed by four node two-dimensional reduced integral bonding element CHO2D4, and the rest of the model is meshed by plane strain four node reduced integral element CEP4R. Figure 16b shows the schematic diagram of finite element meshing of the model.

The finite element models at meso scale and macro scale are established respectively. In order to study the model size effect on fracture toughness, three different sizes are selected for the model at each scale, which are listed in Table 8.

**Table 8:** Specific dimension information of CT model.

Scale	ID	Model size (μm)	Crack length (μm)	Displacement load *U* (μm)	Diameter of pin hole (μm)
Meso scale	1	2.5 × 2.4	1	0.1	0.5
	2	5 × 4.8	2	0.1	1
	3	10 × 9.6	4	0.1	2
Macro scale	4	250 × 240	100	5	50
	5	500 × 480	200	5	100
	6	750 × 720	300	5	150

CZM theory assumes that there is a small cohesion zone at the front of the crack tip, and its mechanical properties obey the TSL. When the cohesion model parameters such as interface strength and cohesion energy are known, the length *L*_*CZ*_ of the cohesion zone can be determined by estimation. For the single mode fracture problem, according to the previous research results^55^, it can be estimated by the following formula:

7 $$L_{CZ} = ME\frac{{G_{IC} }}{{T^{2} }}$$

where, *M* is a parameter, and its value is usually taken as 1, *E* is the transverse elastic modulus, *T* is the interface stress, and *G*_*IC*_ is the critical fracture energy of the material interface. Under the assumption of linear elasticity, the fracture energy of the material has the following relationship with the stress intensity factor *K*:

8 $$G = \frac{{K^{2} }}{{E\left( {1 - \upsilon^{2} } \right)}}$$

where, υ is the Poisson's ratio of the material. Therefore, Eq. (7) can be written as:

9 $$L_{CZ} = ME\frac{{{{K_{IC}^{2} } \mathord{\left/ {\vphantom {{K_{IC}^{2} } {E\left( {1 - \upsilon^{2} } \right)}}} \right. \kern-\nulldelimiterspace} {E\left( {1 - \upsilon^{2} } \right)}}}}{{T^{2} }} = M\frac{{K_{IC}^{2} }}{{\left( {1 - \upsilon^{2} } \right)T^{2} }}$$

In order to ensure the accuracy of numerical simulation, Turon et al.^56^ believe that the length of cohesive element shall not be greater than the length of cohesive zone. In the research of song et al.^57^, it is further pointed out that under macro conditions, when there are 3–5 cohesive elements in the cohesion area, the accuracy requirements can be met. Assuming that the cohesive zone is equally divided into *N*_*e*_ cohesive elements, the length *L*_*e*_ of cohesive elements is calculated as:

10 $$L_{e} = \frac{{L_{cz} }}{{N_{e} }} = M\frac{{K_{IC}^{2} }}{{N_{e} \left( {1 - \upsilon^{2} } \right)T^{2} }}$$

Based on Eq. (10), the length of cohesive element under plane strain can be estimated. The interface strength of four terminal models is obtained from the previous MD calculation. Here, we take the maximum value *T* = 11.7345 GPa, *M* = 1.0, *υ* = 0.3, *N*_*e*_ = 5 in Table 7. Generally speaking, the macroscopic critical interface stress intensity factor of iron *K*_IC_ ∈ [6 MPa·m^1/2^, 20 MPa·m^1/2^]^58^, so the length of cohesive element can be estimated as:

11 $$L_{e} = \frac{{L_{cz} }}{{N_{e} }} = M\frac{{K_{IC}^{2} }}{{N_{e} \left( {1 - \upsilon^{2} } \right)T^{2} }}{ = }\frac{{K_{IC}^{2} }}{{4.55T^{2} }} \in \left[ {0.057\;{{\upmu m, 0}}{.637}\;{\upmu m}} \right]$$

Under the condition of ensuring the calculation accuracy and efficiency, the length of coherent element in macro scale is determined as 0.5 μ m.

### Determination of cohesive material parameters

Our research group conducted experimental and simulation research on the fracture behavior of X80 gas transmission pipeline steel in the early stage^59^. The results show that the calculation results of software embedded bilinear CZM are in good agreement with the experimental data. Therefore, the bilinear CZM is used for α-Fe/Fe_3_C interface crack propagation behavior simulation in this paper. The initial damage stiffness *K*, interface stress *T* and critical fracture energy *G*_*IC*_ of cohesive element must be determined in the process of simulating crack propagation by using of the bilinear CZM. It has been determined the interface stress *T* and critical fracture energy *G*_*IC*_ of α-Fe/Fe_3_C interface by MD simulation, therefore, it is also necessary to determine its initial damage stiffness *K*.

**Figure 17:**
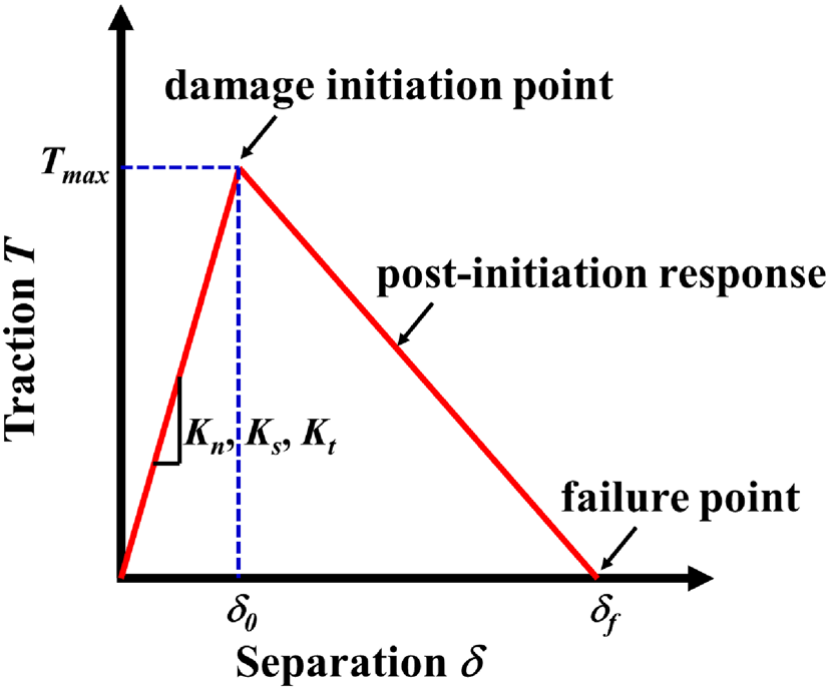
Schematic diagram of triangular TSL.

For the ideal cohesive fracture behavior, the initial damage stiffness *K* defaults to an infinite value, so as to ensure the integrity of the model before element failure. However, the infinite *K* will make the calculation difficult to converge in the actual simulation process, so a large *K* exceeding the threshold is generally set to ensure the stability of the calculation. For the bilinear CZM selected in this work, its TSL is triangular, as shown in Fig. 17. The stiffness in the elastic stage *K*_*e*_ can be determined by the initial damage displacement *δ*_0_, end failure displacement *δ*_*f*_ and critical fracture energy *G*_*IC*_ are obtained, and the calculation formula is:

12 $$K_{e} = \frac{K}{{T_{0} }} = \frac{{2G_{IC} }}{{\delta_{0} \delta_{f}^{{}} }}$$

13 $$K = \frac{{2G_{IC} }}{{\delta_{0} \delta_{f} }}T_{0}$$

where, *K* is the stiffness; *T*_0_ is the initial constitutive calculation thickness of the cohesive element specified by the user, and its value is generally set to 1.

For a single type of fracture, isotropy can be assumed, then the isotropic initial damage stiffness *K*_*n*_ = *K*_*s*_ = *K*_*t*_ = *K*, and the triangular area surrounded by TSL is the critical fracture energy *G*_*IC*_:

14 $$G_{IC} { = }\frac{1}{2}\delta_{f} T_{\max }$$

It can be obtained from Eqs. (13) and (14):

15 $$K = K_{n} = K_{s} = K_{t} = \frac{{T_{\max } }}{{\delta_{0} }}$$

In the simulation process, the maximum nominal stress criterion is selected as the failure criterion of cohesive element, and the isotropic nominal stress is assumed *σ*_*n*_ = *σ*_*s*_ = *σ*_*t*_ = *T*_*max*_, the energy form is selected for the evolution mode of softening stage, and the viscosity coefficient is set to 1e^-5^ to ensure the calculation convergence. Table 9 shows the simulation results of four terminal types of interface fracture extracted by MD method.

**Table 9:** MD simulation results.

Terminal type	Maximum interface stress *T*_*max*_ (GPa)	Initial damage displacement *δ*_0_ (nm)	Critical fracture energy *G*_*IC*_ (μN/μm)	Initial damage stiffness *K* (μN/μm^3^)	Elastic modulus *E* (MPa)
C–Fe	10.327	0.105	6.393	98,993,621.020	296,380.000
Fe–Fe	8.398	0.081	4.980	104,044,541.200	300,630.000
Fe–C	9.977	0.119	6.254	83,914,402.360	293,830.000
C–C	11.735	0.122	6.771	97,756,543.760	300,790.000

**Figure 18:**
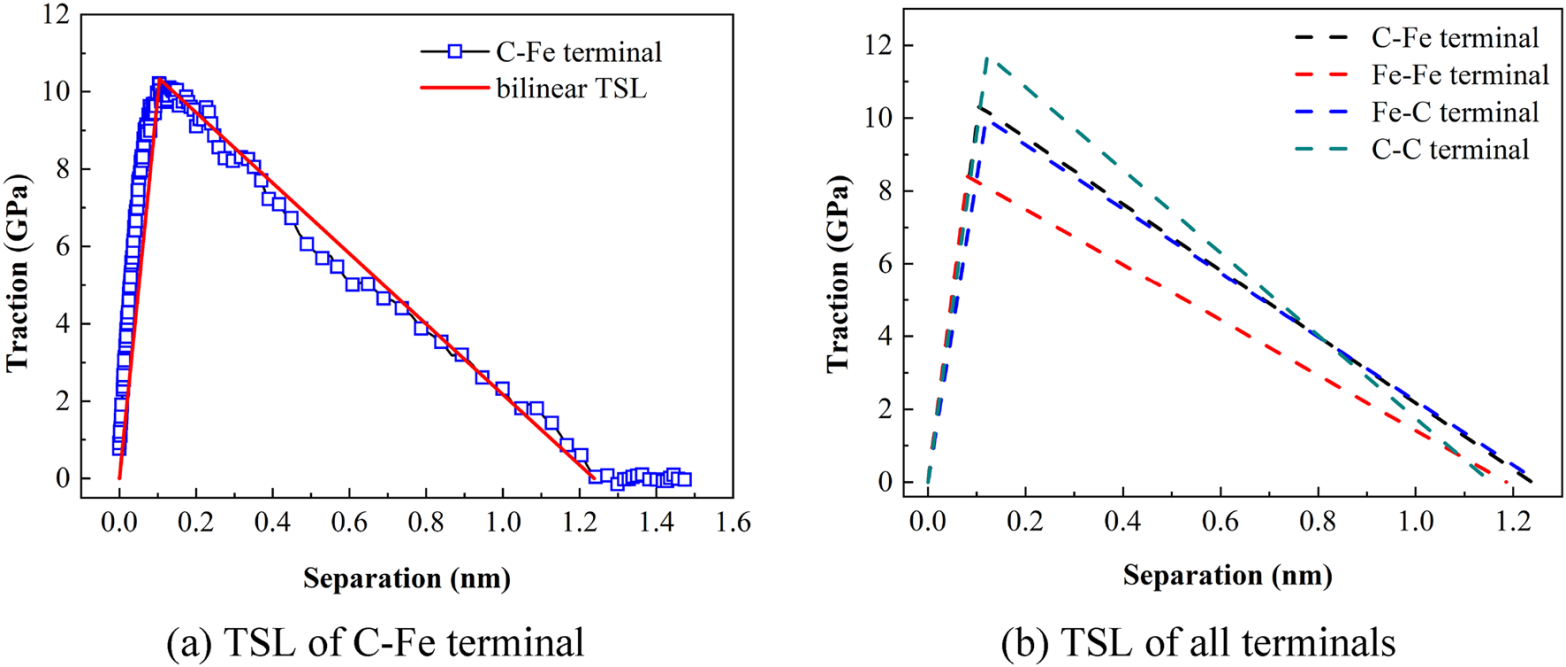
Triangular failure criteria for four terminal type interfaces.

According to Fig. 17 and Table 9, the failure criterion of the bilinear CZM is established. Figure 18a shows the relationship between the triangular failure criterion of the C–Fe terminal interface and the interface TS curve at the atomic scale. Figure 18b shows the triangular failure criteria for all terminal models.

### Simulation results at macro and meso scales

#### Macro scale analysis

##### Analysis of crack initiation state of cohesive element at crack tip

**Figure 19:**
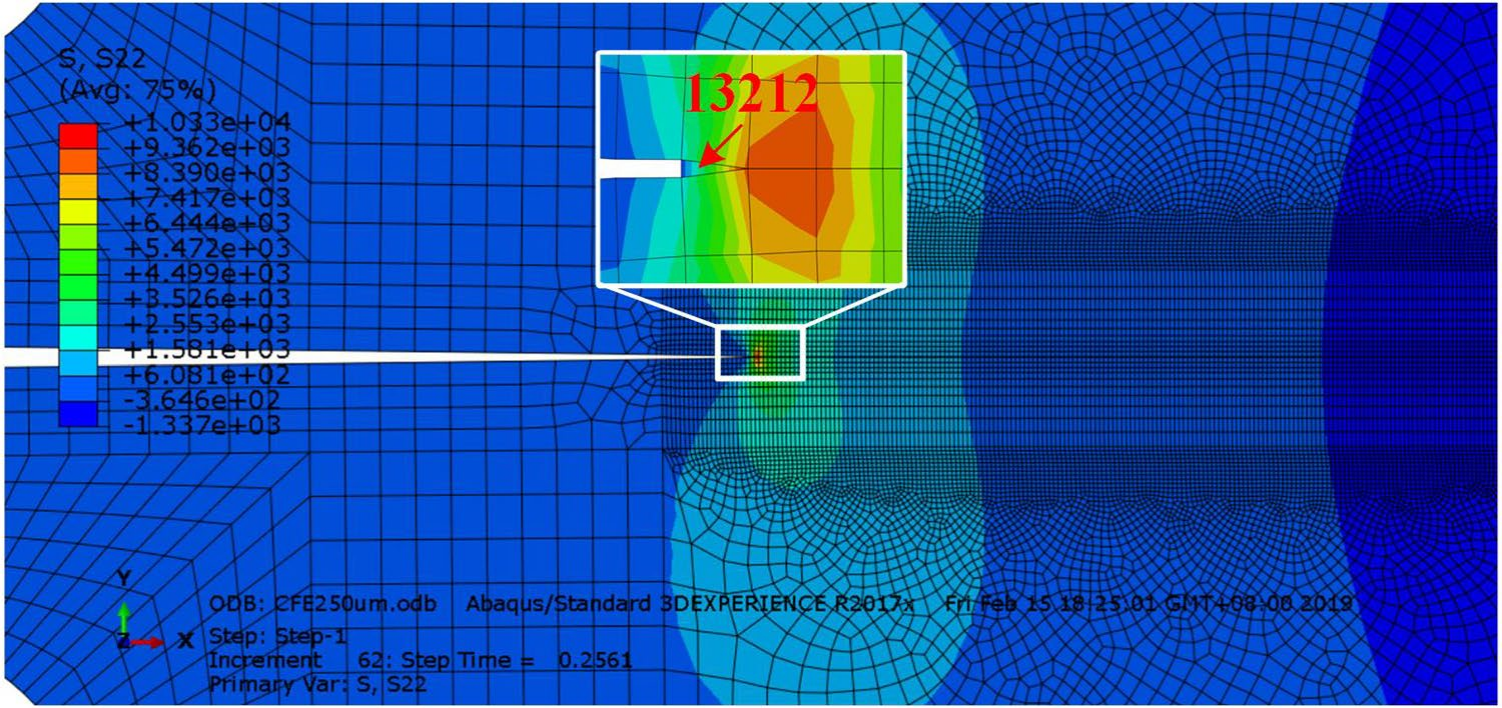
Schematic diagram of 13212 cohesive unit at crack tip.

The crack propagation of material is an instantaneous behavior. When the external load reaches the crack initiation state, the crack will expand rapidly and lead to material failure. Therefore, it is very important to investigate the crack initiation state of cohesive element at the crack tip. Take 250 μm × 240 μm finite element mode of C-Fe terminal as an example, Fig. 19 shows the cloud diagram of instantaneous Mises stress distribution before the failure of cohesive element at the crack tip, and element 13212 is the cohesive element at the crack tip of the model.

**Figure 20:**
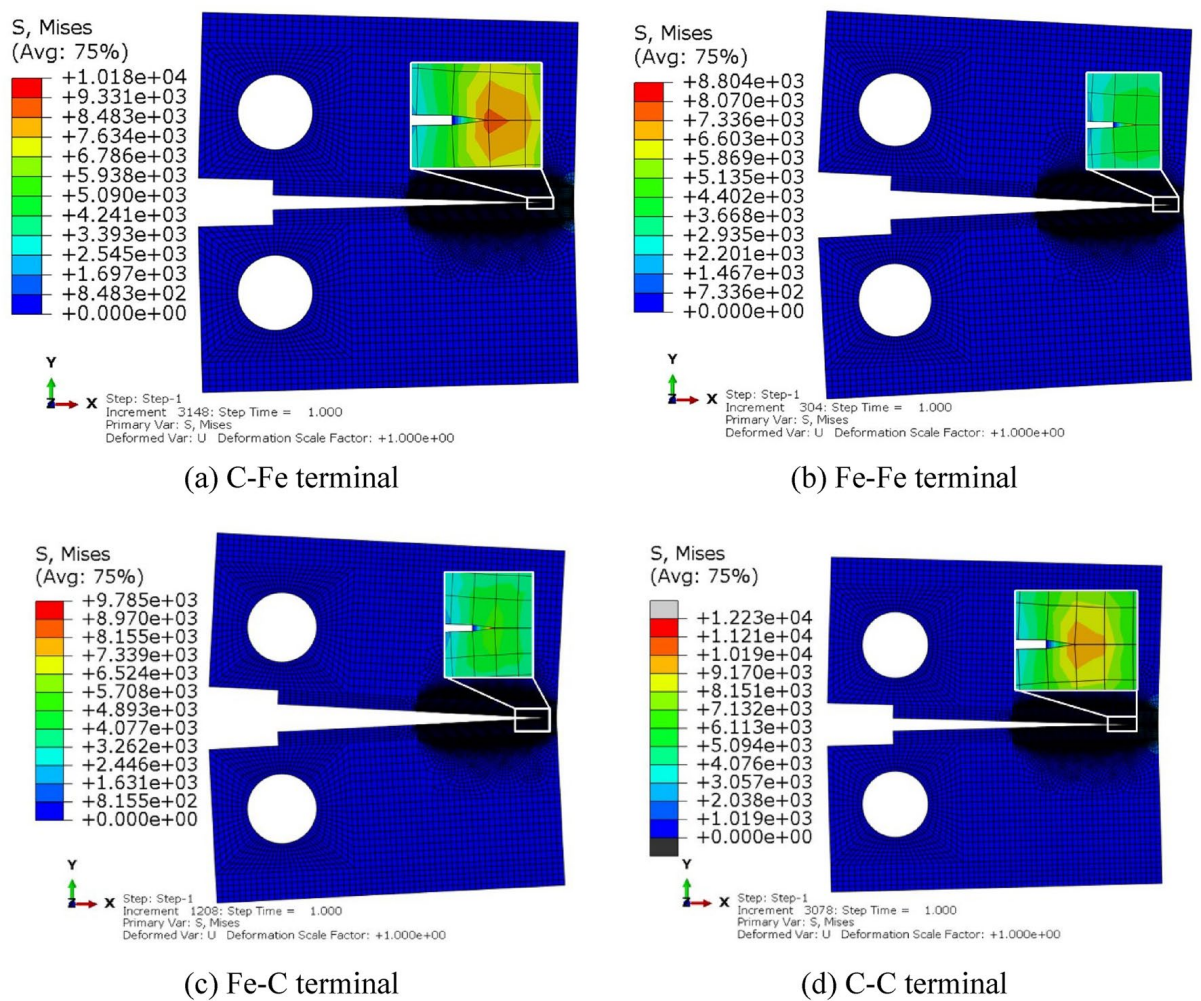
Stresses cloud diagram of different terminal with model size of 250 μm × 240 μm.

For the embedded bilinear CZM, the finite element code sets a STATUS variable in the STEP module to control the destruction and deletion of the element, which is the same as the failure criteria of the trapezoidal CZM and exponential CZM, but when the value of this variable changes from 1 to 0, the element is destroyed and deleted, Therefore, the evolution process of the state variable with loading time can be extracted to determine the crack initiation time. Figure 20 shows the cloud diagram of equivalent stress distribution after loading of 250 μm × 240 μm model.

**Figure 21:**
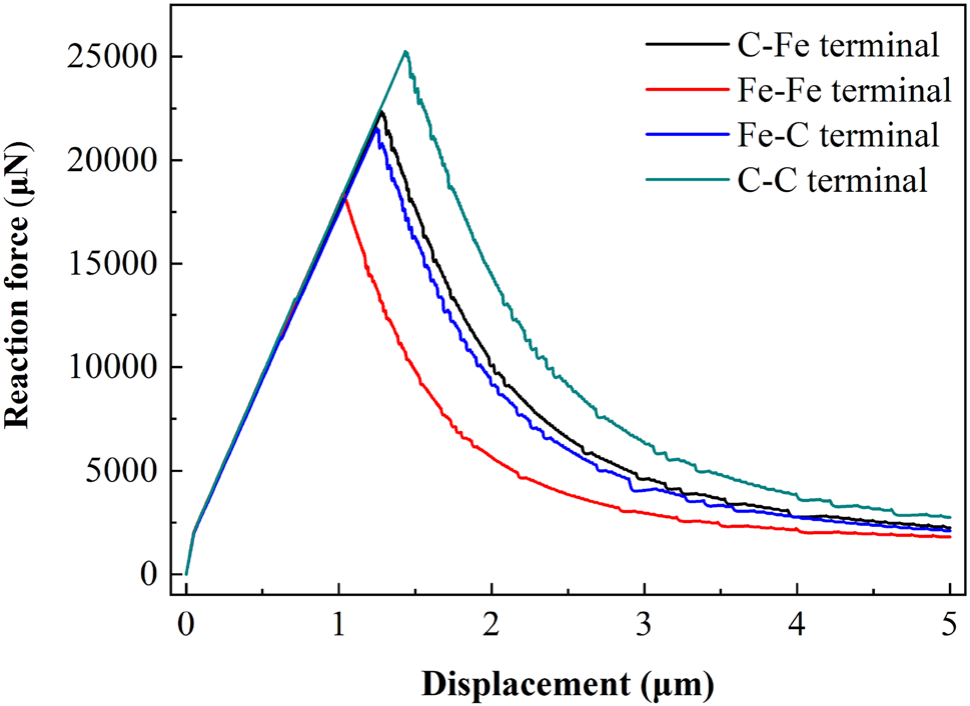
Displacement vs reaction force at loading point (250 μm × 240 μm).

**Figure 22:**
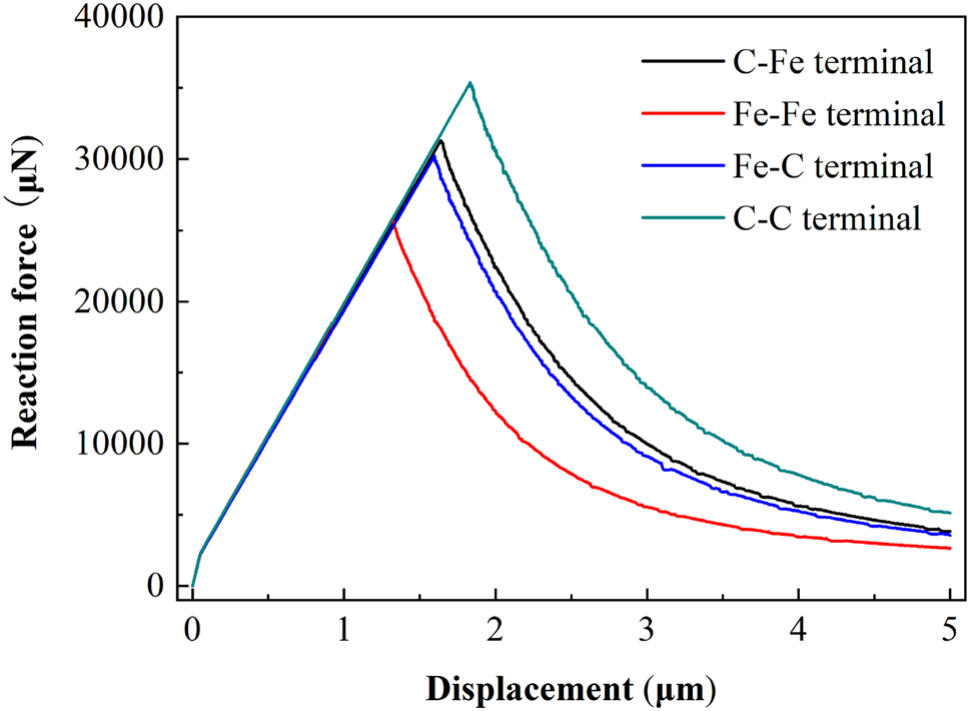
Displacement vs reaction force at loading point (500 μm × 480 μm).

**Figure 23:**
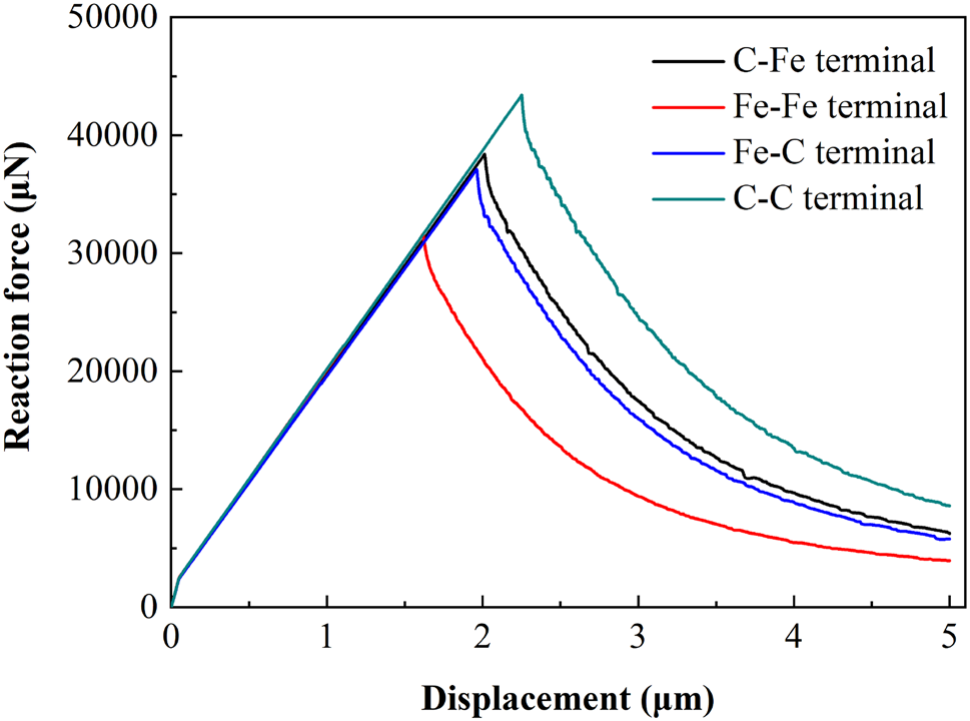
Displacement vs reaction force at loading point (750 μm × 720 μm).

##### Load displacement analysis

For CT specimens, the fracture toughness parameters are obtained from the load displacement curve of the loading point. In the simulation process, the support reaction force and displacement value in the vertical direction of the reference point RP-1 are extracted as the load displacement curve of the loading point. Similarly, for different models, this method is used to extract the load displacement curve of the loading point and determine its limit load and displacement value.

Figures 21, 22 and 23 show the response curves of reaction force and displacement at loading points corresponding to different size models. It can be found that when the reaction force reaches the maximum value, the required external load decreases with the crack propagation. Moreover, for the four terminal interface models, the maximum reaction force is C–C > C–Fe > Fe–C > Fe–Fe, this is consistent with the law displayed at the micro scale.

##### Fracture toughness calculation

**Figure 24:**
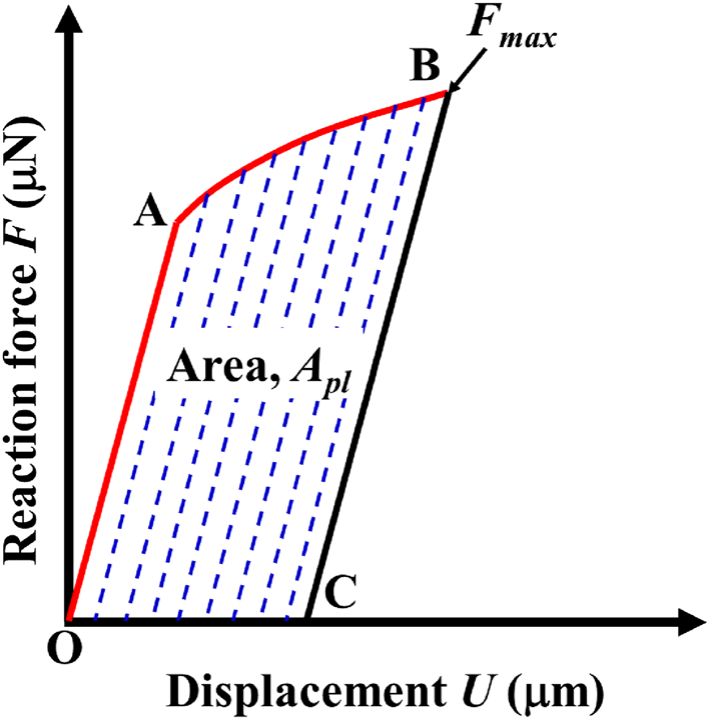
Schematic diagram of plastic work measurement.

**Figure 25:**
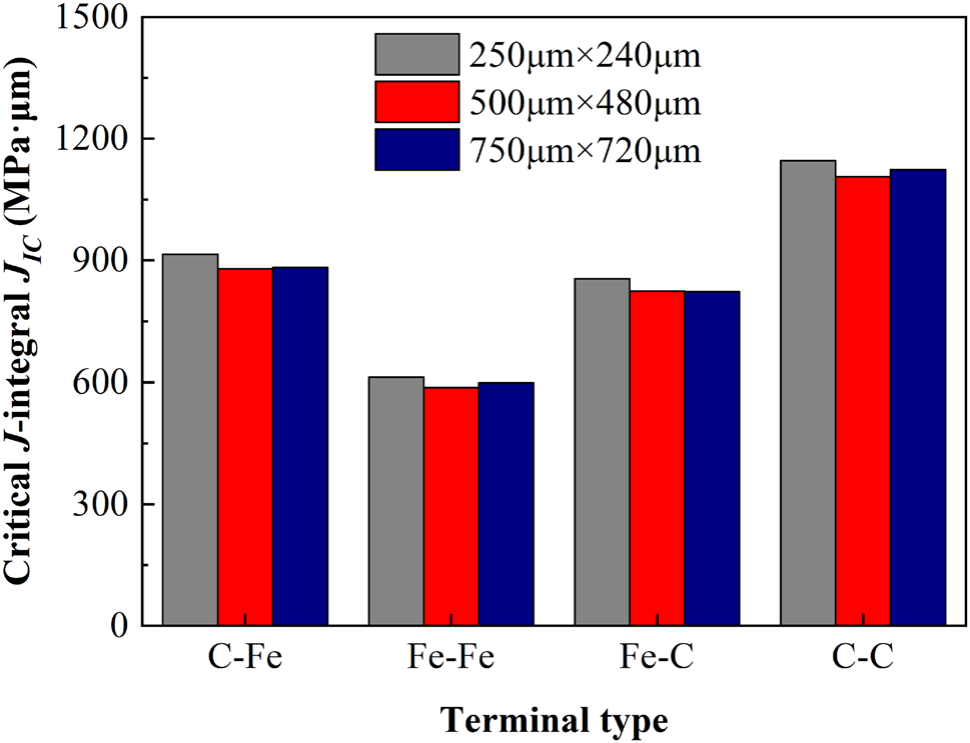
Critical *J*-integral distribution of each terminal interface under different size models.

According to the standard, the calculation formula of critical stress intensity factor *K*_*IC*_ of CT model is:

16 $$K_{IC} = \frac{{F_{i} }}{{\left( {BB_{N} W} \right)^{1/2} }}f\left( {\frac{{a_{i} }}{W}} \right)$$

17 $$f\left( {\frac{{a_{i} }}{W}} \right) = \frac{{\left\{ {\left( {2 + \frac{{a_{i} }}{W}} \right)\left[ {0.886 + 4.64\left( {\frac{{a_{i} }}{W}} \right) - 13.32\left( {\frac{{a_{i} }}{W}} \right)^{2} + 14.72\left( {\frac{{a_{i} }}{W}} \right)^{3} - 5.6\left( {\frac{{a_{i} }}{W}} \right)_{4} } \right]} \right\}}}{{\left( {1 - \frac{{a_{i} }}{W}} \right)^{{{3 \mathord{\left/ {\vphantom {3 2}} \right. \kern-\nulldelimiterspace} 2}}} }}$$

where, *a*_*i*_ is consistent with the dimension of a in Fig. 16a. For the 2D model, the specimen thickness *B* and its net thickness *B*_*N*_ are both taken as 1.0, ignoring the influence of thickness. The calculation formula of critical *J*-integral is:

18 $$J_{IC} = \frac{{K_{IC}^{2} \left( {1 - \upsilon^{2} } \right)}}{E} + J_{pl}$$

19 $$J_{pl} = \frac{{\eta_{pl} A_{pl} }}{{B_{N} b_{o} }}$$

where, *J*_*pl*_ is the plastic part of *J*-integral; *η*_*pl*_ is the plasticity factor, *η*_*pl*_ = 2 + 0.522*b*_*o*_/*W*; *b*_*o*_ is the length of residual ductile zone, *b*_*o*_ = *W* − *a*; *A*_*pl*_ is the plastic work. The plastic work *A*_*pl*_ can be obtained according to the displacement vs reaction force curve of the loading point. As shown in Fig. 24, OAB is the displacement vs force curve of the loading point. Point B is the limit load, and the parallel line of OA passing through point B intersects with the abscissa at point C, then the area enclosed by OABC is the plastic work *A*_*pl*_. Therefore, the critical stress intensity factor and *J*-integral of each terminal interface model under different sizes can be obtained from Table 9 and Figs. 21, 22 and 23. The calculation results are given in Table 10 and Fig. 25.

**Table 10:** Results of fracture toughness calculated by finite element method of different models at macro scale.

Model size (μm)	Terminal type	ID	Maximum support reaction *F*_max_ (μN)	Displacement at the maximum support reaction *U* (μm)	Critical stress intensity factor *K*_*IC*_ (MPa·μm^1/2^)	Critical J-integral *J*_*IC*_ (MPa·μm)
250 × 240	C–Fe	1	22,335.800	1.281	15,255.227	914.888
	Fe–Fe	2	18,394.000	1.027	12,563.000	611.666
	Fe–C	3	21,546.900	1.248	14,716.413	855.234
	C–C	4	25,247.000	1.435	17,243.561	1145.914
500 × 480	C–Fe	5	31,308.600	1.639	15,120.488	879.569
	Fe–Fe	6	25,753.200	1.316	12,437.508	587.707
	Fe–C	7	30,218.400	1.596	14,593.976	824.331
	C–C	8	35,142.100	1.844	16,971.877	1106.495
750 × 720	C–Fe	9	38,390.900	2.013	15,138.569	882.845
	Fe–Fe	10	31,582.400	1.617	12,453.794	598.540
	Fe–C	11	37,077.000	1.962	14,620.463	822.387
	C–C	12	43,391.600	2.250	17,110.480	1123.499

It can be seen from Table 10 that the critical stress intensity factor is distributed at 12,563.000 MPa·μm^1/2^–17,243.561 MPa·μm^1/2^, the experimental test results of steel are 2000 MPa·μm^1/2^ ~ 60000 MPa·μm^1/2^^60^, it can be seen that the simulation results are within a reasonable range. At the same time, the critical *J*-integral distribution of ferrite–pearlite interface at this scale is 611.67 MPa·μm ~ 1145.91 MPa·μm and the calculated value sequenced by C–C > C–Fe > Fe–C > Fe–Fe. As can be seen from Fig. 25, the calculated *J*_*IC*_ values of the four terminal interfaces under the three size models are very close, and the relative error is small. Therefore, it is considered that the size effect of the calculated results under this scale is small.

#### Meso scale analysis

##### (1) Sizing of cohesive element

**Figure 26:**
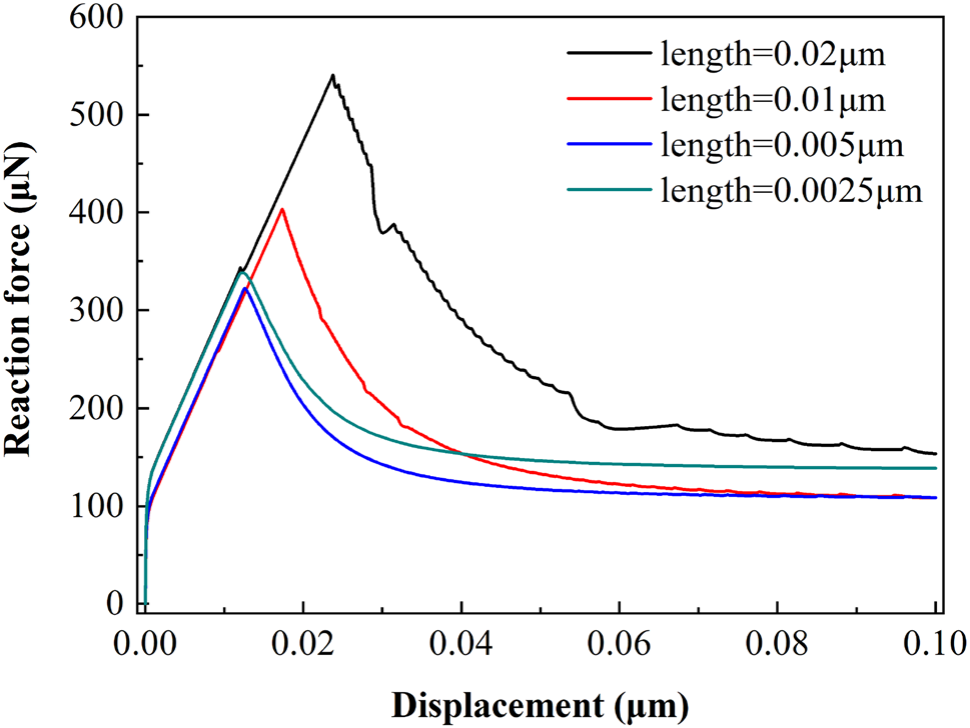
Displacement vs reaction force curves of cohesive elements with different characteristic length.

At the meso scale, the interfacial fracture toughness of materials is difficult to be obtained by experiments, so the characteristic size of cohesive element cannot be estimated directly. Therefore, it is necessary to analyze the sensitivity of grid density at the meso scale. From Eq. (9) and Table 9, it can be calculated that the effective length of cohesion zone of the four terminal models is 0.01479 μm, 0.01766 μm, 0.01846 μm and 0.02127 μm, respectively. Take the C-Fe terminal model with size of 2.5 μm × 2.4 μm as an example, the characteristic lengths of four cohesive elements are meshed respectively, and the applied displacement load is 0.1 μm. The results include force vs displacement curve of RF-1 can be found in Table 11 and Fig. 26.

**Table 11:** length sensitivity analysis results of cohesive element.

Model size (μm)	ID	Coherent element size (μm)	Total number of elements	Maximum support reaction *F*_max_ (μN)	Displacement at the maximum support reaction *U* (μm)
2.5 × 2.4	1	0.020	4924	518.456	0.025
	2	0.010	7851	403.268	0.017
	3	0.005	14,916	322.014	0.013
	4	0.0025	31,143	338.264	0.012

**Figure 27:**
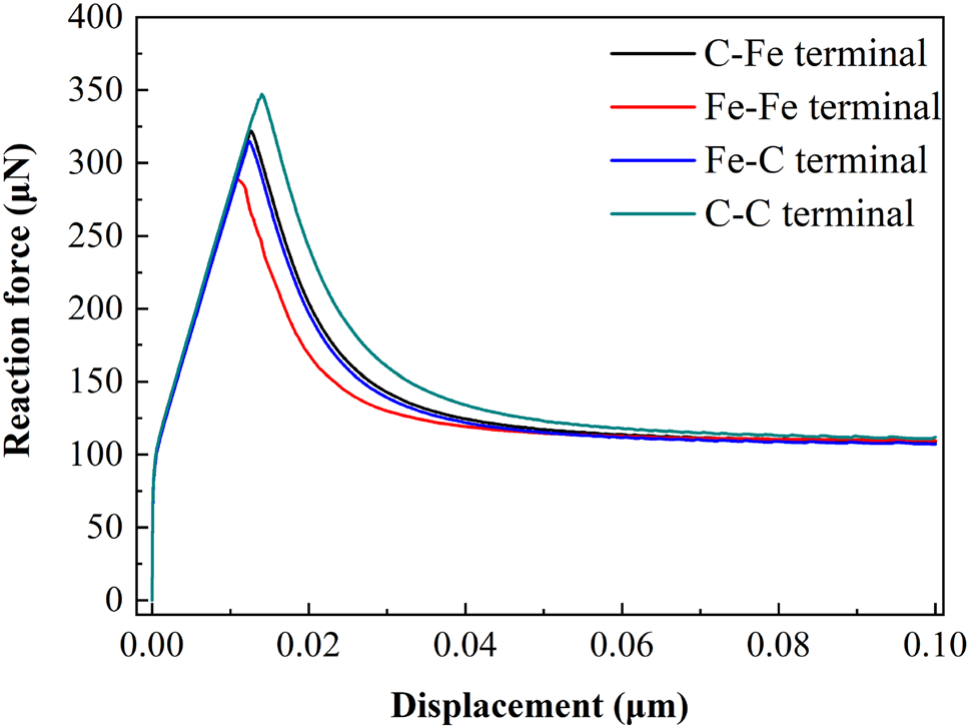
Displacement vs reaction force at loading point (2.5 μm × 2.4 μm).

**Figure 28:**
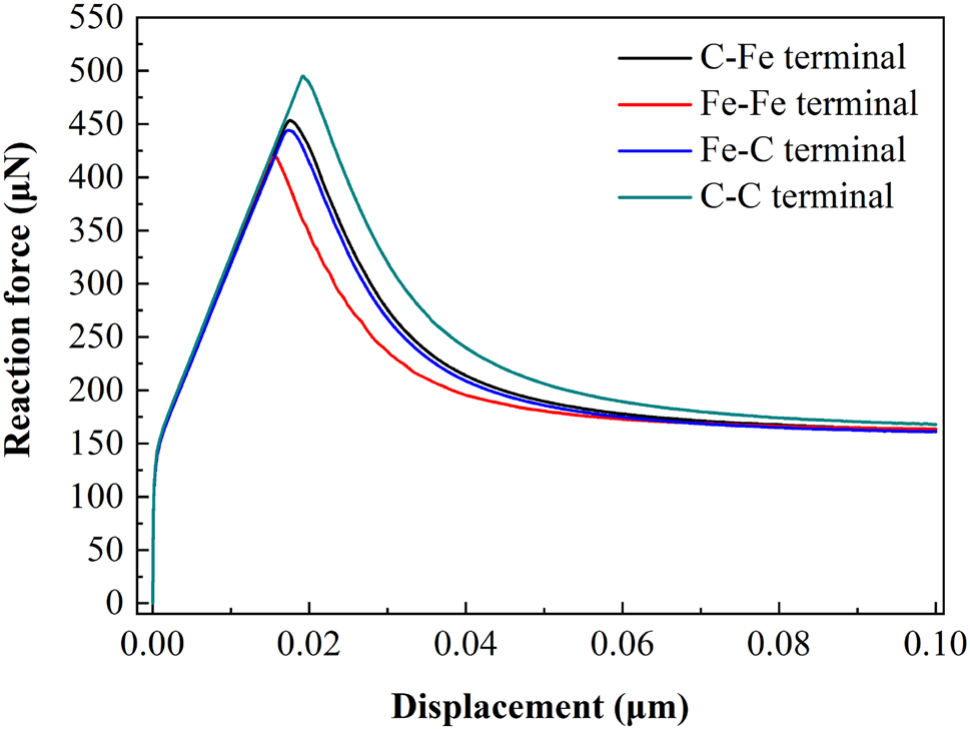
Displacement vs reaction force at loading point (5 μm × 4.8 μm).

**Figure 29:**
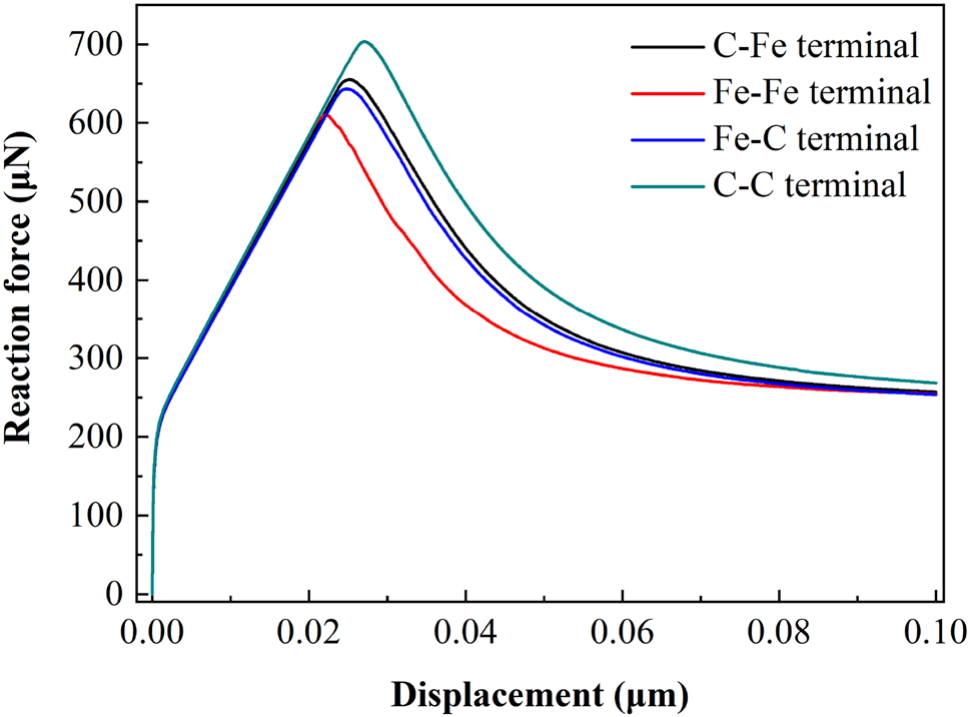
Displacement vs reaction force at loading point (10 μm × 8 μm).

The results in Fig. 26 show that when the characteristic length of cohesive element is 0.005 μm, the maximum reaction force *F*_max_ does not decrease. When the mesh density is further subdivided into 0.0025 μm, the maximum support reaction force *F*_max_ and the corresponding displacement are very close to the element with characteristic length of 0.005 μm. Considering that the calculation efficiency can be improved as much as possible under the condition of accuracy, the characteristic length of cohesive element at meso scale is taken as 0.005 μm.

##### (2) Analysis of simulation results

By using the model parameters in Table 11, the cracking process of CT model is simulated. Displacement vs reaction force curves are drawn in Figs. 27, 28 and 29. It can be found that the law is consistent with the finite element simulation results at macro scale, but the maximum reaction force is much smaller than the macro results.

##### (3) Fracture toughness calculation

**Figure 30:**
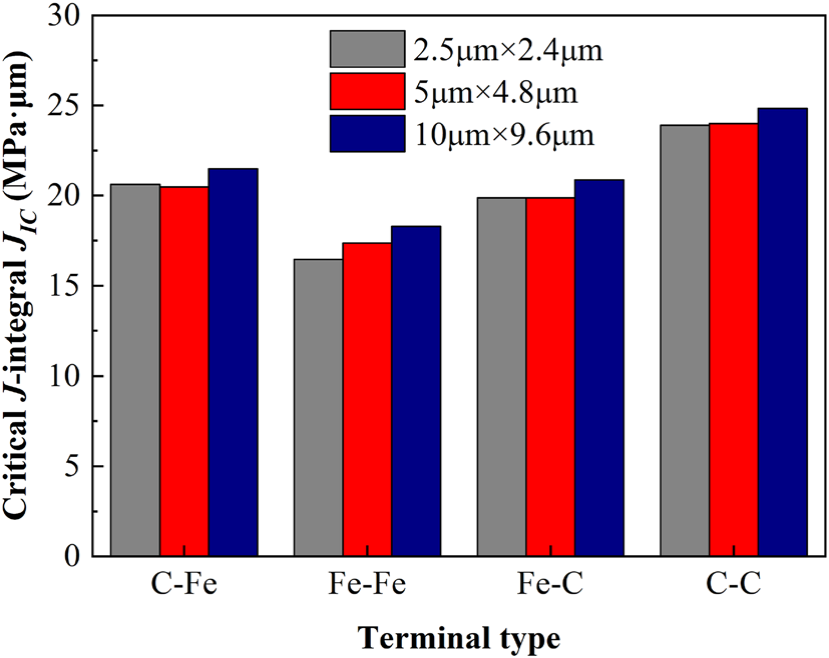
Critical *J*-integral distribution of each terminal interface under different size models.

Based on the above theory, the fracture toughness obtained by finite element simulation at the micro scale is shown in Table 12 and Fig. 30. It is found that the critical *J*-integral distribution of ferrite–pearlite interface at this scale is 16.446–24.822 MPa·μm and their value sequence is C–C > C–Fe > Fe–C > Fe–Fe. It can be seen from Fig. 30 that the *J*_*IC*_ values calculated by the four terminal interfaces under the three size models are very close, and the relative error is small. Therefore, it is considered that the size effect of the calculation results under this scale is much less, which is like the models with macro size.

**Table 12:** Fracture toughness results calculated by finite element method of different models at micro scale.

Model size (μm)	Terminal type	ID	maximum support reaction *F*_max_ (μN)	Displacement at the maximum support reaction *U* (μm)	Critical stress intensity factor *K*_*IC*_ (MPa·μm^1/2^)	Critical J-integral *J*_*IC*_ (MPa·μm)
2.5 × 2.4	C–Fe	1	322.014	0.013	2199.338	20.613
	Fe–Fe	2	289.329	0.011	1976.101	16.446
	Fe–C	3	314.945	0.012	2151.057	19.860
	C–C	4	347.225	0.014	2371.527	23.908
5 × 4.8	C–Fe	5	453.188	0.018	2188.671	20.467
	Fe–Fe	6	419.869	0.016	2027.757	17.354
	Fe–C	7	444.103	0.017	2144.795	19.867
	C–C	8	494.871	0.019	2389.979	23.996
10 × 9.6	C–Fe	9	655.081	0.025	2237.083	21.487
	Fe–Fe	10	610.247	0.022	2083.977	18.273
	Fe–C	11	643.184	0.025	2196.455	20.860
	C–C	12	703.355	0.027	2401.938	24.822

## Conclusion

Based on serial multi-scale analysis method, molecular dynamics and finite element cohesion model are combined to realize the transmission of cohesion parameters from bottom-up to macro and meso scales at atomic scale. The critical *J*-integral *J*_*IC*_ of type I cracking calculated by extracting the displacement and support reaction curves at the loading point of macro and micro size models, whose value is 611.670–1145.910 MPa·μm and 16.446–24.822 MPa·μm, respectively. The model size effect can be ignored in both micro scale and wire macro scale. The results show that the serial multi-scale analysis method is feasible to judge the crack propagation behavior of high strength and toughness pipeline steel.

According to the plane type of cementite terminal, the mechanical parameters such as maximum interface stress and cohesion energy at different scales are: C–C > C–Fe > Fe–C > Fe–Fe, which shows that the promotion of C atom on the strength and toughness of pipeline steel can be considered when studying the crack arrest performance of high strength and toughness pipeline steel. As the simulation scale increases from nano scale → micron scale → silk meter scale → centimeter scale, the critical *J*-integral *J*_*IC*_ also increases step by step. Through multiscale analysis, the parameters characterizing the internal crack propagation mechanism of pipeline steel at the atomic scale combined with the CZM can be transferred to the macro scale, providing a new and effective way to judge the crack initiation characteristics of high strength and toughness pipeline steel pipe for engineering practice.

However, with the continuous progress of pipe rolling technology, the fracture resistance of pipeline steel has been greatly improved with the addition of more trace elements in higher API steel grades. Therefore, it is very necessary to further study the micro fracture properties of pipeline steel with chemical elements, which is also one of the topics that our group will pay attention to in the next step.

## Data Availability

The datasets generated during and/or analysed during the current study are not publicly available due to confidentiality requirements of project research but are available from the corresponding author on reasonable request.
